# Aluminum-Centered C–C Heterocoupling of Organonitriles

**DOI:** 10.1021/acs.organomet.5c00335

**Published:** 2025-10-10

**Authors:** Alannah C. M. Thomas, Estelle M. Bouchat, Kyle G. Pearce, Louis J. Morris, Rex S. C. Charman, Michael S. Hill

**Affiliations:** Department of Chemistry, 1555 University of Bath, Claverton Down, Bath BA2 7AY, U.K.

## Abstract

The *C-tert-*butyl substituted azacyclopropenylaluminate, [(SiN^Dipp^)­Al-η^2^-{NC*t*-Bu}­K]_∞_ (SiN^Dipp^ = (CH_2_SiMe_2_NDipp)_2_) reacts with both aryl- and alkyl-substituted nitriles. In contrast to previously reported attempts to effect C–C bond formation between two dissimilar nitriles, these reactions enable the completely discriminating heterocoupling of *t*-BuCN to provide diazabutadienylaluminate dianions that bear two differentiated N=*C* substituents.

## Graphical abstract

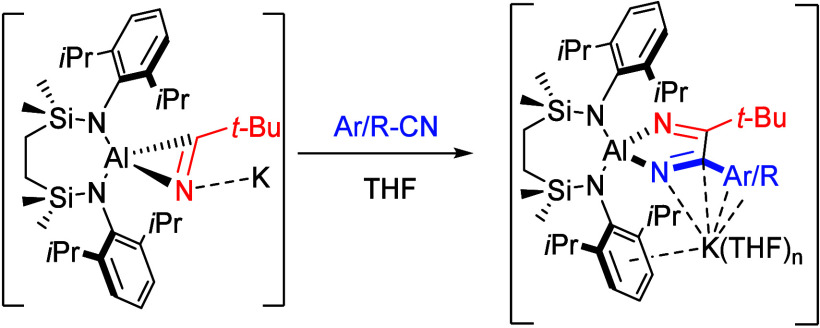

Novel means to generate new C–C bonds from readily available substrates are a defining objective of organic chemistry. In this regard, metal-centered transformations of unsaturated organic molecules, whether using an s-, p-, d-, or f-element centered reagent,^1–6^ have been productively exploited since the onset of modern chemical synthesis. Directed by availability and sustainability considerations, many recent advances have focused on the more Earth-abundant metals with, for example, iron to the fore as a potential alternative to longer established precious metal reagents.^7,8^ Derived from an electropositive element representing *ca*. 8% of the Earth’s crust, aluminum compounds capable of selective *C*-element bond formation, thus, present especially attractive targets for development. Although commonly constrained to its more thermodynamically favored +3 state, the strongly reducing behavior of aluminum in lower (+2, +1) oxidation states holds particular appeal for the activation of small organic molecules.^9,10^ A case in point is provided by the Al­(I) centers of diamidoalumanyl anions, the stability and reactivity of which were first demonstrated by Aldridge and Goicoechea’s pivotal report of [KAl­(_xanth_NON)]_2_ (_xanth_NON = 4,5-(NDipp)_2_-2,7-*t*-Bu_2_-9,9-Me_2_-xanthene; Dipp = 2,6-*i*-Pr_2_C_6_H_3_),^11^ and rapidly followed by a plethora of closely related systems.^12–22^ Characteristic of such species, we have recently reported that the potassium alumanyl, [(SiN^Dipp^)­AlK]_2_ (**1**, (SiN^Dipp^ = (CH_2_SiMe_2_NDipp)_2_), reacts readily with organic nitriles, R/ArC≡N (Scheme 1a).^23^ Treatment of compound **1** with either *o*- or *m*-tolunitrile provided C–C coupling and formation of the corresponding diazabutadienylaluminate derivatives, **2** and **3**. A similar outcome was observed with *i-*PrCN, albeit in this case C–C bond formation was accompanied by an unusual 2-fold C–H to N–H isomerization of a *C*-*iso*-propyl substituent to provide the 1-alumina-2,5-diazabutadiene derivative (**4**). This outcome was reasoned to be sterically induced and the impact of increasing nitrile steric demand was further emphasized by reaction of **1** with *t*-BuCN, which resulted in [2 + 1] cycloaddition of the C≡N bond at each Al­(I) center and the selective formation of the potassium azacyclopropenylaluminate derivative [(SiN^Dipp^)­Al-η^2^-{NC*t*-Bu}­K]_∞_ (**5**).

**Scheme 1:**
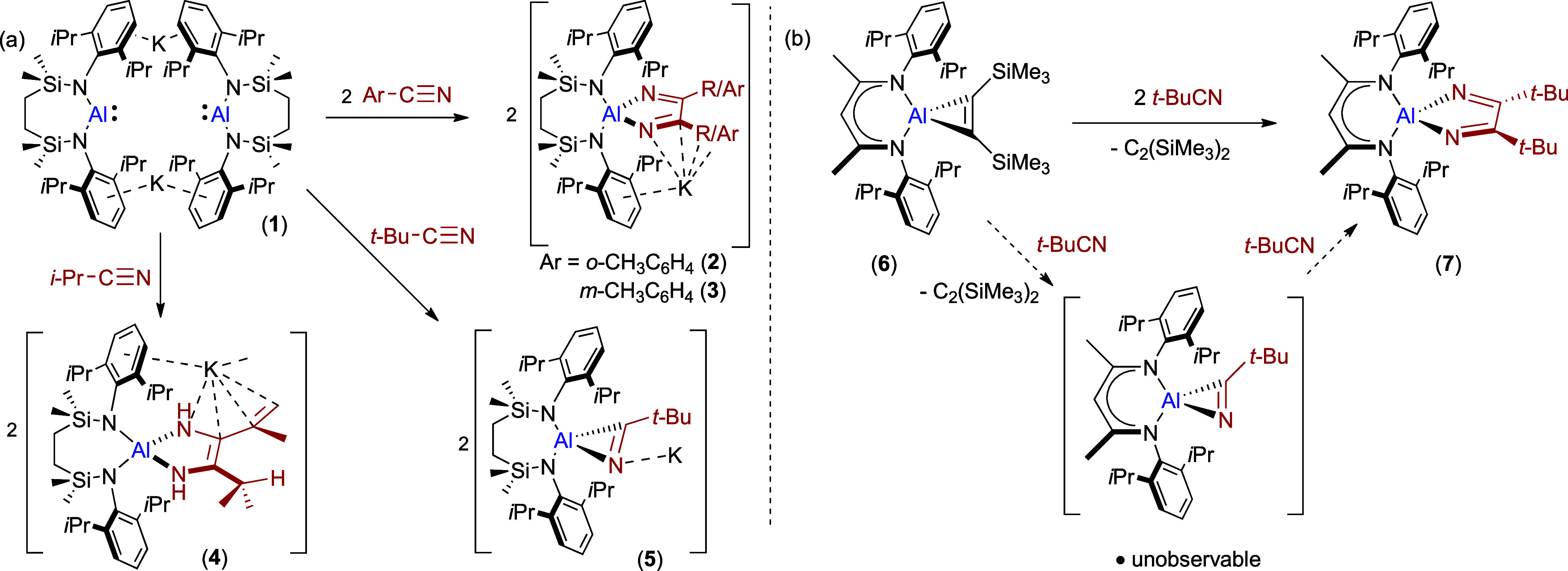
(a) Reactivity of Compound **1** with Organic Nitriles;^23^ (b) Roesky and Co-workers’ Proposed Stepwise Formation of Compound **7** ^24^

The isolation of **5** implies that the formation of compounds **2**–**4** is a stepwise process via the intermediacy of similar, but unobservable, azacyclopropenyl intermediates.^25^ This experimental deduction is reminiscent of Roesky and co-workers’ earlier study of the reactivity of *t*-BuCN with [(BDI)­Al­{η^2^-C_2_(SiMe_3_)_2_}] (**6**; BDI = HC­{(Me)­CNDipp}_2_). (Scheme 1b),^24^ which behaves as an apparent surrogate of the charge neutral Al­(I) β-diketiminate by displacement of Me_3_SiC≡CSiMe_3_. Although reacting with 2 equiv of nitrile to yield **7**, the formation of this diazabutadiene product was similarly proposed to occur via an alumina-azacyclopropene intermediate.

Similar reductive coupling of nitriles has been mediated by several low oxidation state magnesium^27,28^ and rare earth systems.^29,30^ The chemistry summarized in Scheme 1 is, however, and to the best of our knowledge, the sole precedent for such aluminum-centered reactivity. The behavior of **6** (Scheme 1b) is also reminiscent of the significant body of research arising from the nitrile-based reactivity of various Group 4 (Ti, Zr) metallocene bis­(trimethylsilyl)­acetylene complexes,^26,31–35^ which, in a similar manner, proceed through alkyne displacement (Scheme 2). The ultimate C–C coupled products in these cases, however, have been calculated to form by sequential κ^1^-NCR coordination to the Group 4 centers.^31^ The pathway shown in Scheme 2 was supported by the experimental identification of a singly adducted zirconocene-nitrile intermediate, while the various equilibria established are consistent with the nitrile crossover observed when the initial metalladiazabutadiene products are treated with an alternative nitrile (R′CN, Scheme 2). These features of the zirconocene chemistry have been recognized by Reiß and Beweries as a potential means to achieve the heterocoupling of 2-cyanofuran and 2-cyanothiophene.^36^ Irrespective of the order of nitrile addition and a product bias toward the hoped for unsymmetrical chelate, the lability of this system to equilibrate yielded an inseparable mixture of all three possible homo- and heterocoupled products such that a protocol for the selective heterocoupling of nitriles remains to be achieved. Herein, therefore, we report that the ready isolation and notably stable structure of compound **5** enables the direct C–C coupling of *t*-BuCN with dissimilar organic nitriles.

**Scheme 2:**
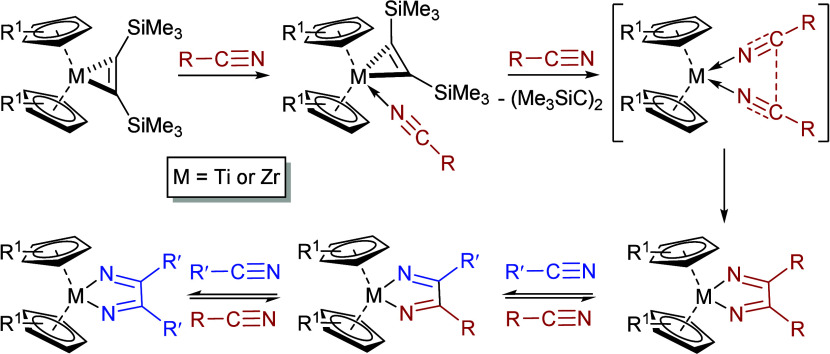
Coupling of Nitriles by Group 4 Metallocene Bis­(trimethylsilyl)­acetylene Complexes^26^

An initial equimolar reaction was performed at room temperature in THF between compound **5** and benzonitrile (Scheme 3). Although analysis of the reaction by ^1^H and ^13^C­{^1^H} NMR spectroscopy was consistent with the instantaneous formation of a single new compound incorporating both reagents, removal of volatiles provided compound **8** as a yellow oil that proved to be resistant to further purification or crystallization. Further reactions of **5** with *o*-, *m-* or *p-*tolunitrile provided a similar outcome, albeit now with the presentation of diagnostic (3H by relative integration) aryl methyl resonances in their respective ^1^H NMR spectra at δ 2.50 (**9**), 2.18 (**10**) and 2.39 (**11**) ppm. Definitive evidence for the constitutions of compounds **8**–**11** was provided by X-ray diffraction analysis of single crystals of compounds **9** and **10**, which were obtained from THF/hexane solvent mixtures.

**Scheme 3:**
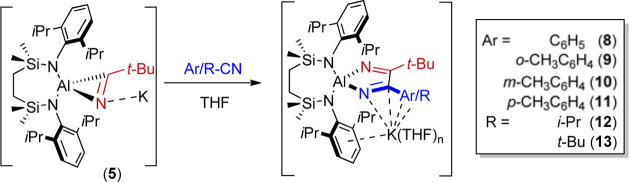
Reactions of Compound **5** with Aryl (Ar) and Alkyl (R) Nitriles and the Syntheses of Compounds **8**–**13**

Although refinement of both data sets was hampered by disorder in the (SiN^Dipp^) and THF ligands, the results of these analyses (Figure 1) provided unambiguous confirmation that compounds **9** and **10** were the heterocoupled diazabutadienylaluminate products. Both compounds crystallize as monomeric contact ion pairs with the potassium (K1) cations encapsulated by a combination of diazabutadiene nitrogen coordination [**9**, 2.814(3); **10**, 2.846­(6) Å] and polyhapto K···π-arene interactions with the Dipp and tolyl substituents. Although K^+^ coordination is completed by either three (**9**) or two (**10**) molecules of THF, the various analogous bond lengths and angles across both structures are closely comparable and consistent with the previously reported data provided by compounds **2** and **3**, which were respectively characterized as a solvent-free molecular dimer and as a bis-THF-coordinated ion pair.^23^

**Figure 1:**
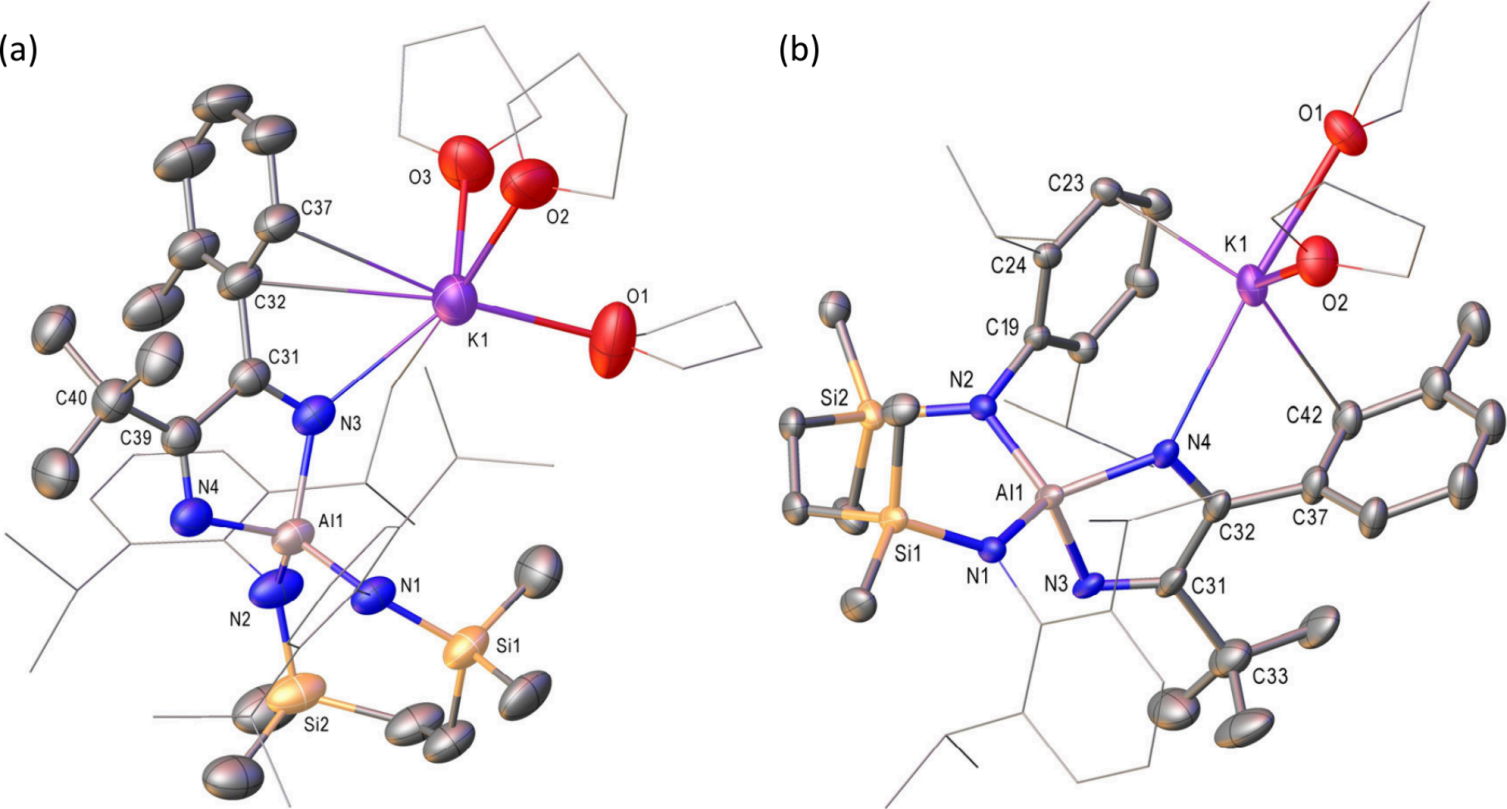
Displacement ellipsoid plots (30% probability) of (a) compound **9** and (b) compound **10**. Hydrogen atoms plus disordered atoms have been removed for clarity. A wireframe view has been employed for some groups, also for visual ease. Selected bond lengths (Å) and angles (°): (**9**) Al1–N1 1.868(3), Al1–N2 1.854(3), Al1–N3 1.908(3), Al1–N4 1.874(3), N3–C31 1.277(4), N4–C39 1.270(4), C31–C39 1.582(5), N2–Al1–N1 112.78(13), N4–Al1–N3 91.63(12); (**10**) Al1–N1 1.867(3), Al1–N2 1.865(3), Al1–N3 1.912(4), Al1–N4 1.856(6), N3–C31 1.234(7), N4–C32 1.281(7), C31–C32 1.545(9), N2–Al1–N1 114.37(11), N4–Al1–N3 92.1(2).

Mindful of *iso*-propyl isomerization during the formation of compound **4** (Scheme 1a), compound **5** was reacted with an equimolar equivalent of *i*-PrCN in THF. Despite the expectation prompted by this immediate precedent, the resultant colorless solution presented a ^1^H NMR spectrum that evidenced the generation of a single new species (**12**). Although these data were again indicative of significant asymmetry across the (SiN^Dipp^) chelate, they also confirmed the integrity of the *tert*-butyl (δ_H_ = 0.69 ppm, s, 9H) and *iso*-propyl substituents originating from both nitrile substrates (Scheme 3). This deduction was ratified by a further X-ray diffraction analysis performed on pale orange single crystals of **12**, which were again isolated from a THF/hexane solvent system (Figure 2a). The structure of compound **12** is broadly analogous to that of the similarly tris-THF K-adducted ion pair **9**. Although the separation between K1 and the closest *N*-donor center of the aluminate anion is only marginally elongated in comprison to either **9** or **10** [*ca*. 2.81–2.84 versus 2.88 Å], the contrasting steric demands of the C32-bonded *iso*-propyl susbtituent result in disruption of the K···π arene interactions that were a common feature of both earlier described structures. The K1 coordination environment is consequently better considered as being provided by a combination of the three THF donors and a variety of C–H···K close contacts to the Dipp- and N=C-*iso*-propyl substituents. Although this structural change otherwise exerts minimal impact upon the tetraazaaluminate structure of **12**, its ready formation highlights that the inaccessibility of a bis *C*-*iso*-propyl-based analog implied by our previous identification of **4** should not be rationalized solely on steric grounds.

**Figure 2:**
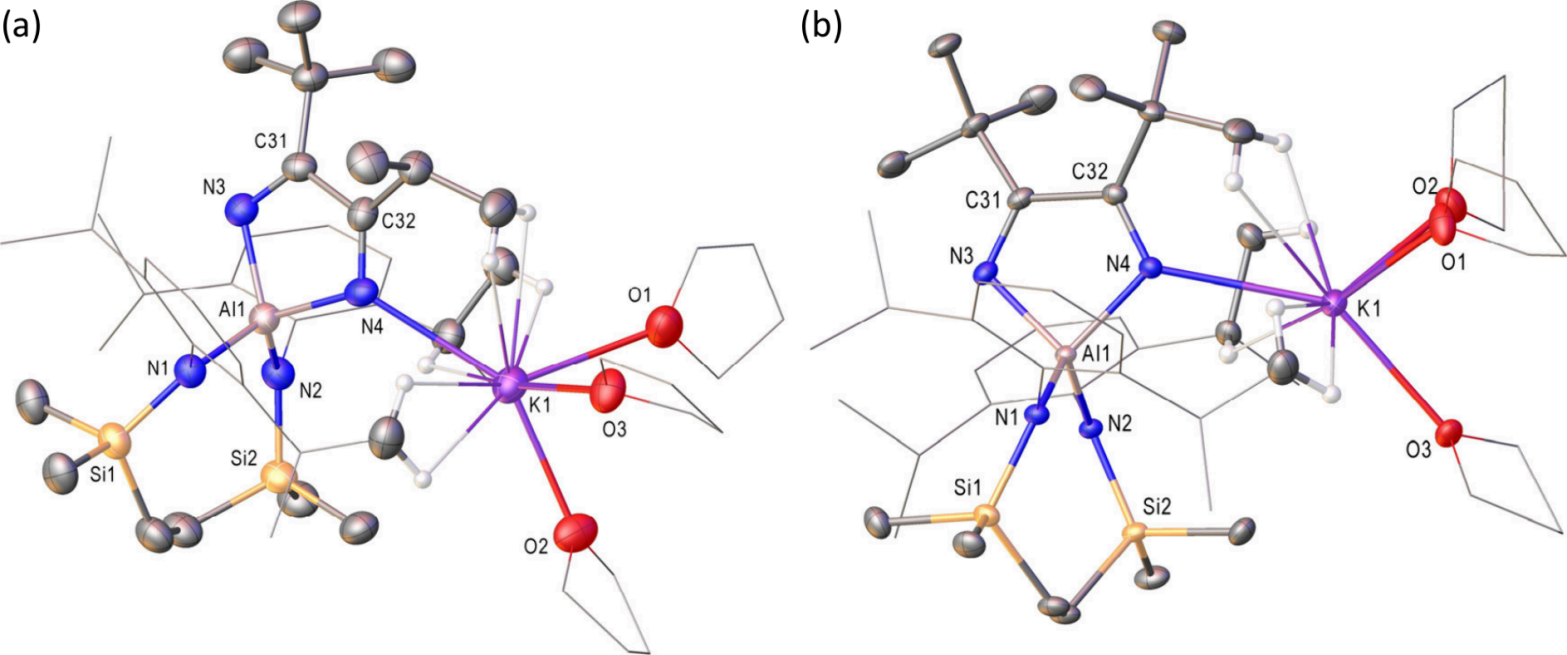
Displacement ellipsoid plots (30% probability) of (a) compound **12** and (b) the Al1-containing molecule of compound **13**. Hydrogen atoms, except those interacting with K1, plus disordered atoms have been removed for clarity. A wireframe view has been employed for some groups, also for visual ease. Selected bond lengths (Å) and angles (°): (**12**) Al1–N1 1.907(3), Al1–N2 1.856(3), Al1–N3 1.865(3), Al1–N4 1.872(3), N3–C31 1.277(5), N4–C32 1.282(5), C31–C32 1.581(5), N2–Al1–N1 111.84(14), N4–Al1–N3 92.80(14); (**13**) Al1–N1 1.8673(10), Al1 N2 1.8632(10), Al1–N3 1.8636(10), Al1–N4 1.9075(10), N3–C31 1.2639(16), N4–C32 1.2728(15), C31–C32 1.6332(17), N2–Al1–N1 112.50(4), N3–Al1–N4 92.10(5).

Although we have not further explored the processes resulting in the isolation of **4**, as a further assessment of the impact of increasing nitrile steric demands we re-examined the reaction of **5** with a equimolar equivalent of *t*-BuCN in THF (Scheme 3).^23^ While no evidence of reaction was again observed at room temperature, overnight heating of the initial pale yellow solution at 60 °C provided a colorless solution, analysis of which by ^1^H NMR spectroscopy indicated the generation of a single product (**13**), most clearly characterized by a singlet resonance assigned to a *t* *ert*-butyl environment with a relative intensity of 18H at δ_H_ 0.72 ppm. The implied process of nitrile insertion and the formation of the (NC*-t*-Bu)_2_ diazabutadienylaluminate chelate was confirmed by a single crystal X-ray diffraction analysis of **13** (Figure 2b). Although the asymmetric unit of **13** comprises two independent molecules, their structures are effectively identical and, obviating any further neceassary comment, analogous to that of the similarly tris-THF-ligated potassium *C-*dialkyldiazabutadienylaluminate (**12**).

In conclusion, the ready isolation and stability of the *C-tert-*butyl substituted azacyclopropenylaluminate **5** allows its further reaction with both aryl- and alkyl-nitriles. In contrast to previously reported attempts to effect C–C bond formation between two dissimilar nitriles, these reactions enable the selective heterocoupling of *t*-BuCN to provide diazabutadienyl dianions that bear two differentiated *C*-substituents and present no indication of the nitrile equilibration that limited the chemistry summarized in Scheme 2. We are continuing to study the reactivity of **5** and related compounds to effect selective aluminum-centered transformations.

## Supplementary Material



## Notes

Details of procedures relevant to the synthesis of compounds **8**–**13**, NMR spectra, and a summary of the X-ray diffraction experiments (PDF)
