# Supplementary material for: Aluminum-Centered C–C Heterocoupling of Organonitriles
Source: Organometallics. 2025 Oct 10;44(20):2369–73. doi: 10.1021/acs.organomet.5c00335 (PMC12570258; doi:10.1021/acs.organomet.5c00335)
Supplement: Supplementary file 1 [file om5c00335_si_001.pdf]

Supporting Information for

Aluminum-centered C-C Heterocoupling of Organonitriles

Alannah C. M. Thomas, Estelle M. Bouchat, Kyle G. Pearce, Louis J. Morris, Rex. S. C. Charman and Michael S. Hill*

Department of Chemistry, University of Bath, Claverton Down, Bath, BA2 7AY, United Kingdom

Email: msh27@bath.ac.uk

Table of Contents

Synthesis S2

General Considerations S2

Synthesis of [{SiN^{Dipp}}$_2$Al(*t*-BuCN)(C_6H_5CN)K] (**8**) S2

Synthesis of [{SiN^{Dipp}}$_2$Al(*t*-BuCN)(*o*-$CH_3C_6H_4$CN)K(THF)$_3$] (**9**) S2

Synthesis of [{SiN^{Dipp}}$_2$Al(*t*-BuCN)(*m*-$CH_3C_6H_4$CN)K(THF)$_2$] (**10**) S3

Synthesis of [{SiN^{Dipp}}$_2$Al(*t*-BuCN)(*p*-$CH_3C_6H_4$CN)K(THF)$_3$] (**11**) S3

Synthesis of [{SiN^{Dipp}}$_2$Al{(*t*-BuCN)(C_3H_7CN)}K] (**12**) S3

Synthesis of [{SiN^{Dipp}}$_2$Al(*t*-BuCN)$_2$K] (**13**) S4

NMR Spectra S5

X-ray Crystallography S20

Table S1. Crystal Data and Structure Refinement for Compounds **9**, **10**, **12** and **13** S21

References S22

Synthesis

General Considerations. Unless stated otherwise, all experiments were conducted using standard Schlenk line and/or glovebox techniques under an inert atmosphere of argon. NMR spectra were recorded with a Bruker Avance III spectrometer (^{1}H at 400 MHz, ^{13}C at 101 MHz). The spectra are referenced relative to residual protio solvent resonances. Elemental analyses were performed at London Metropolitan University. Solvents were dried by passage through a commercially available solvent purification system and stored under argon in ampoules over 4 Å molecular sieves. d_8-THF was purchased from Sigma-Aldrich and dried over a potassium mirror before distillation and storage over molecular sieves. Compound **5** was prepared according to the reported procedures.[1] Organic nitriles were purchased from Merck and degassed by three freeze-pump-thaw cycles and stored over 4 Å molecular sieves for more than 18 hours prior to usage.

Synthesis of [{SiN^{Dipp}}$_2$Al(t-BuCN)(C$_6$H$_5$CN)K] (8)

In a J Young's NMR tube compound **5** (20 mg, 0.031 mmol) was dissolved in d_8-THF (~0.5 mL), after which benzonitrile (3.2 µL, 0.031 mmol) was added to the yellow solution. Volatiles were removed *in vacuo* to provide a dark yellow oil. Attempted crystallization by using variations of a hexane and THF solvent system was unsuccessful and compound **8** (27.9 mg, 97%) was, thus, obtained as a dark yellow oil. ^{1}H NMR (400 MHz, d_8-THF) δ_H: 7.72 – 7.47 (m, 5H, Ar-H), 7.10 – 6.69 (m, 6H, Dipp-Ar-H), 4.05 (m, 4H, Dipp-CHMe$_2$), 1.25 – 1.08 (m, 24H, Dipp-CHMe$_2$), 0.97 (s, 4H, SiCH$_2$), 0.52 (s, 9H, *t*-Bu CH$_3$), 0.09 (s, 6H, SiMe$_2$), –0.01 (s, 6H, SiMe$_2$) ppm. ^{13}C{^{1}H} NMR (101 MHz, d_8-THF) δ_C: 174.7 (*C*=N), 174.1 (*C*=N), 150.7 (Ar*C*), 148.6 (Ar*C*), 148.1 (Ar*C*), 146.8 (Ar*C*), 133.6 (Ar*C*), 133.1 (Ar*C*), 130.2 (Ar*C*), 128.4 (Ar*C*), 127.6 (Ar*C*), 126.3 (Ar*C*), 123.8 (Ar*C*), 122.7 (Ar*C*), 121.6 (Ar*C*), 113.9 (Ar*C*), 40.4 (*t*-Bu*C*), 31.9 (*C*H$_3$-*t*-Bu), 28.1, 27.9, 27.8, 27.6, 26.7 (*C*HMe$_2$), 25.4, 16.3, 1.6 (Si*C*H$_3$), 1.0 (Si*C*H$_3$) ppm. Multiple attempts to obtain a satisfactory CHN elemental analysis were unsuccessful.

Synthesis of [{SiN^{Dipp}}$_2$Al(t-BuCN)(o-CH$_3$C$_6$H$_4$CN)K(THF)$_3$] (9)

In a J Young's NMR tube Compound **5** (20 mg, 0.031 mmol) was dissolved in d_8-THF (~0.5 mL), after which *o*-tolunitrile (3.67 µL, 0.031 mmol) was added to the deep yellow solution. Upon addition of the nitrile the solution turned pale yellow, volatiles were removed *in vacuo* and the pale-yellow solid was redissolved in THF and layered with hexane. This provided dark yellow crystals of compound **9** (29.5 mg, 99%) suitable for X-ray diffraction analysis. ^{1}H NMR (400 MHz, d_8-THF) δ_H: 7.64 – 7.26 (m, 4H, Ar-H), 6.98 – 6.60 (m, 6H, Dipp-Ar-H), 4.44 (s, 1H, Dipp-CHMe$_2$), 4.15 (m, 1H, Dipp-CHMe$_2$), 4.06 (m, 1H, Dipp-CHMe$_2$), 3.81 (s, 1H, Dipp-CHMe$_2$), 2.50 (s, 3H, *o*-CH$_3$), 1.25 – 1.03 (m, 24H, Dipp-CHMe$_2$), 1.00 (s, 4H, SiCH$_2$), 0.52 (s, 9H, *t*-Bu CH$_3$), 0.09 (s, 6H, SiCH$_3$), -0.01 (s, 6H, SiCH$_3$) ppm. ^{13}C{^{1}H} NMR (101 MHz, d_8-THF) δ_C: 174.4 (N*C*), 174.0 (N*C*), 153.2 (Ar*C*), 151.4 (Ar*C*), 148.1 (Ar*C*), 147.7 (Ar*C*), 142.7 (Ar*C*), 133.6 (Ar*C*), 133.3 (Ar*C*), 131.2 (Ar*C*), 127.4 (Ar*C*), 130.0 (Ar*C*), 127.1

(Ar*C*), 125.2 (Ar*C*), 124.3 (Ar*C*), 114.1 (Ar*C*), 40.0 (*t*-Bu), 29.9 (*t*-Bu-*C*H$_3$), 28.1, 27.5, 27.1, 26.8, 26.7, 25.4, 20.5 (*o*-CH$_3$), 16.5 (Si*C*H$_2$), 1.7 (Si*C*H$_3$), 1.0 (Si*C*H$_3$) ppm. Elemental analysis: $C_{55}H_{90}AlKN_4O_3Si_2$ (calculated as tris-THF adduct) requires C, 67.6; H, 9.3; N, 5.7%; found C, 69.3; H, 6.8; N, 5.5%.

Synthesis of [{SiN^{Dipp}}$_2$Al(t-BuCN)(m-CH$_3$C$_6$H$_4$CN)K(THF)$_2$] (10)

In a J Young's NMR tube compound **5** (20 mg, 0.031 mmol) was dissolved in d$_8$-THF (~0.5 mL), after which *m*-tolunitrile (3.68 µL, 0.031 mmol) was added to the yellow solution. Volatiles were removed *in vacuo* and the dark yellow oil was crystallized by layering a THF solution with hexane. Compound **10** (27.3 mg, 94%) was obtained as pale yellow crystals. ^{1}H NMR (400 MHZ, d$_8$-THF) δ_H: 7.54 – 7.29 (m, 4H, Ar-H), 7.00 – 6.59 (m, 6H, Dipp-Ar-H), 4.05 (m, 4H, Dipp-CHMe$_2$), 2.18 (s, 3H, *m*-CH$_3$), 1.23 – 1.09 (m, 24H, Dipp-CHMe$_2$), 1.06 (s, 4H, SiCH$_2$), 0.53 (s, 9H, *t*-Bu CH$_3$), 0.09 (s, 6H, SiMe$_2$), -0.01 (s, 6H, SiMe$_2$) ppm. ^{13}C{^{1}H} NMR (101 MHz, d$_8$-THF) δ_C: 175.0 (N*C*), 174.2 (N*C*), 153.3 (Ar*C*), 150.8 (Ar*C*), 148.7 (Ar*C*), 146.7 (Ar*C*), 140.5 (Ar*C*), 134.4 (Ar*C*), 133.3 (Ar*C*), 130.1 (Ar*C*), 128.9 (Ar*C*), 128.2 (Ar*C*), 127.0 (Ar*C*), 123.9 (Ar*C*), 122.7 (Ar*C*), 121.6 (Ar*C*), 113.7 (Ar*C*), 40.5 (*t*-Bu), 30.7 (*t*-Bu *C*H$_3$), 28.1, 27.9, 27.7, 27.5, 26.7, 25.6, 21.2 (*m*-CH$_3$), 16.3 (Si*C*H$_2$), 1.7 (Si*C*H$_3$), 1.6 (Si*C*H$_3$) ppm. Elemental analysis: $C_{51}H_{82}AlKN_4O_2Si_2$ (calculated as bis-THF adduct) requires C, 67.7; H, 9.1; N, 6.2%; found 68.6; H, 9.0; N, 6.7%.

Synthesis of [{SiN^{Dipp}}$_2$Al(t-BuCN)(p-CH$_3$C$_6$H$_4$CN)K(THF)$_3$] (11)

In a J Young's NMR tube Compound **5** (20 mg, 0.031 mmol) was dissolved in THF (~0.5 mL), after which *p*-tolunitrile (3.63 mg, 0.031 mmol) was added to the yellow solution. Volatiles were removed *in vacuo* providing a yellow oil. Attempted crystallization from various combinations of THF and hexane were unsuccessful and compound **11** (26.7 mg, 92%) was thus obtained as a yellow oil. ^{1}H NMR (400 MHz, d$_8$-THF) δ_H: 7.59 – 7.29 (m, 4H, Ar-H), 6.96 – 6.69 (m, 6H, Dipp-Ar-H), 4.05 (m, 4H, Dipp-CHMe$_2$), 2.39 (s, 3H, *p*-CH$_3$), 1.25 – 1.08 (m, 24H, Dipp-CHMe$_2$), 1.07 (s, 4H, SiCH$_2$), 0.52 (s, 9H, *t*-Bu CH$_3$), 0.09 (s, 6H, SiMe$_2$), –0.01 (s, 6H, SiMe$_2$) ppm. ^{13}C{^{1}H} NMR (101 MHz, d$_8$-THF) δ_C: 174.4 (N*C*), 173.3 (N*C*), 150.9 (Ar*C*), 148.7 (Ar*C*), 148.1 (Ar*C*), 146.7 (Ar*C*), 144.6 (Ar*C*), 132.9 (Ar*C*), 130.9 (Ar*C*), 129.2 (Ar*C*), 127.6 (Ar*C*), 123.9 (Ar*C*), 121.7 (Ar*C*), 110.8 (Ar*C*), 40.5 (*t*-Bu), 30.7 (*t*-Bu *C*H$_3$), 28.1, 27.8, 27.7, 27.6, 26.8, 25.6, 21.9 (*p*-CH$_3$), 16.3 (SiH$_2$), 1.6 (Si*C*H$_3$), 1.0 (Si*C*H$_3$) ppm. Elemental analysis: $C_{51}H_{82}AlKN_4O_2Si_2$ (calculated as bis-THF adduct) requires C, 67.7; H, 9.1; N, 6.2%; found C, 66.0; H, 8.2; N, 5.5%.

Synthesis of [{SiN^{Dipp}}$_2$Al{(t-BuCN)(C$_3$H$_7$CN)}K] (12)

In a J Young's NMR tube compound **5** (20.2 mg, 0.031mmol) was dissolved in d$_8$-THF (~0.5 mL), after which *i*-PrCN (2.82 µL, 0.031 mmol) was added to the yellow solution. Upon addition of the nitrile the

solution turned colorless. Volatiles were removed *in vacuo* and the pale yellow solid was redissolved in THF and layered with hexane to provide pale orange crystals of compound **12** (23.6 mg, 85%) suitable for X-ray diffraction analysis. ^{1}H NMR (400 MHz, d_8-THF) δ_H: 7.01 – 6.49 (m, 6H, Dipp-H), 4.10 (m, 4H, Dipp-CHMe$_2$), 2.47 (hept, J = 8 Hz, 1H, CHMe$_2$), 1.36 – 1.02 (m, 24H, Dipp-CHMe$_2$), 0.97 (s, 4H, SiCH$_2$), 0.69 (s, 9H *t*-Bu CH$_3$), 0.49 (s, 6H, CHMe$_2$), 0.09 (s, 6H, SiMe$_2$), -0.01 (s, 6H, SiMe$_2$) ppm. ^{13}C{^{1}H} NMR (101 MHz, d_8-THF) δ_C: 176.1 (N*C*), 174.4 (N*C*), 151.4 (Ar*C*), 150.4 (Ar*C*), 148.1 (Ar*C*), 147.5 (Ar*C*), 123.8 (Ar*C*), 122.7 (Ar*C*), 122.0 (Ar*C*), 120.8 (Ar*C*), 39.4 (*t*-Bu), 35.8 (*i*Pr*C*H), 30.4 (*t*-Bu *C*H$_3$), 28.1, 27.7, 27.5, 27.5, 26.7, 25.4, 26.2, 16.6 (Si*C*H$_2$), 1.5 (Si*C*H$_3$), 1.0 (Si*C*H$_3$) ppm. Multiple attempts to obtain a satisfactory CHN elemental analysis were unsuccessful.

Synthesis of [{SiN^{Dipp}}$_2$Al(t-BuCN)$_2$K] (13)

In a J Young's NMR tube Compound **5** was dissolved in d_8-THF (~0.5 mL) forming a pale yellow solution, after which *t*-BuCN (2.0 µL, 0.018 mmol) was. The reaction was heated at 60° overnight added causing the solution to turn colourless. Volatiles were removed *in vacuo* leaving a colorless solid, which was crystallized using hexane and several drops of THF. This provided colorless crystals of Compound **13** (8 mg, 47%) suitable for X-ray diffraction analysis. ^{1}H NMR (400 MHz, d_8-THF) δ_H: 6.96 – 6.50 (m, 6H, Dipp-H), 4.46 (s, 2H, Dipp-CHMe$_2$), 4.10 (m, 2H, Dipp-CHMe$_2$), 1.25 – 1.06 (m, 24H, Dipp-CHMe$_2$), 0.99 (s, 4H, SiCH$_2$), 0.72 (s, 18H, *t*-BuCH$_3$), -0.01 (s, 12H, SiMe$_2$) ppm. ^{13}C{^{1}H} NMR (101 MHz, d_8-THF) δ_C: 177.7 (N*C*), 150.6 (Ar*C*), 148.1 (Ar*C*), 122.8 (Ar*C*), 120.8 (Ar*C*), 41.1 (*t*-Bu), 31.9 (*t*-Bu*C*H$_3$), 27.5, 26.0, 16.0 (Si*C*H$_2$), 1.7 (Si*C*H$_3$) ppm. Elemental analysis: $C_{44}H_{76}AlKN_4OSi_2$ (calculated as mono-THF adduct) requires C, 66.1; H, 9.6; N, 7.0%; found C, 66.3; H, 10.3; N, 6.3%.

NMR Spectra

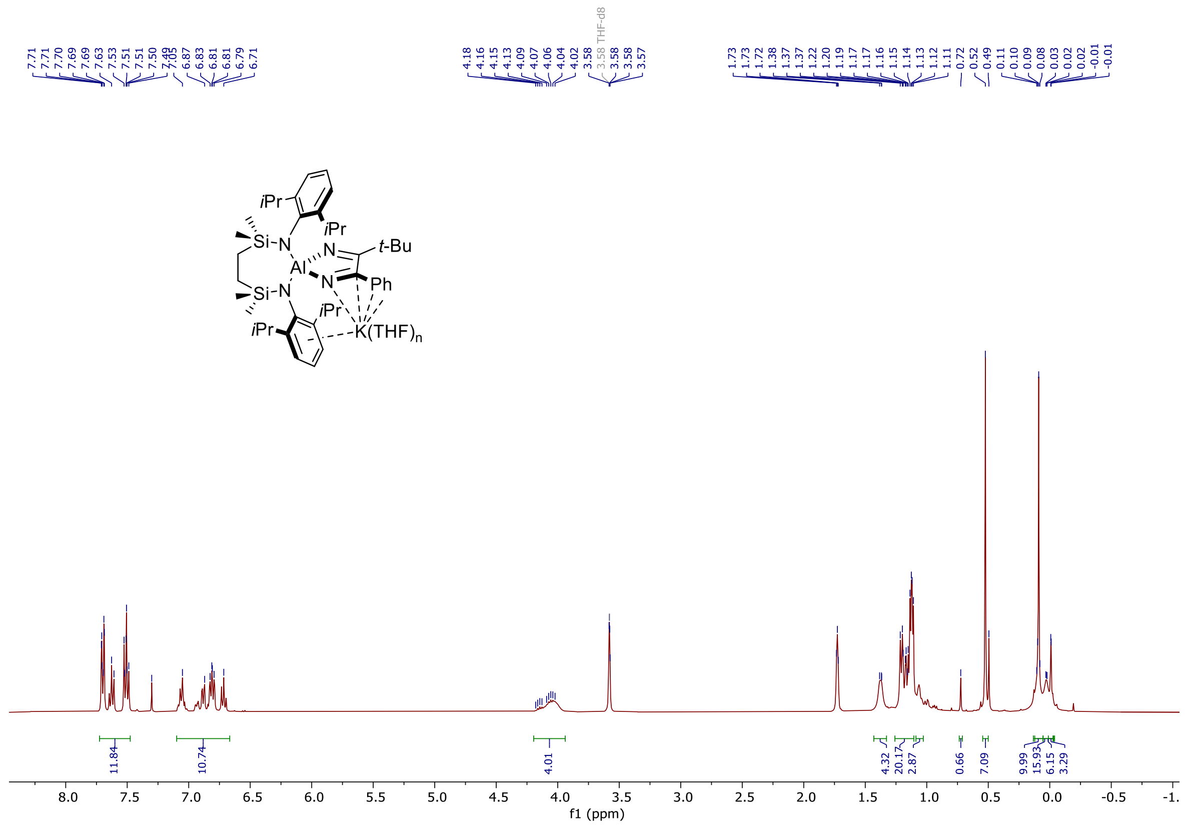

Figure S1: *^{1}H NMR (400 MHz, 298 K, d₈-THF) spectrum of Compound* ***8****.*

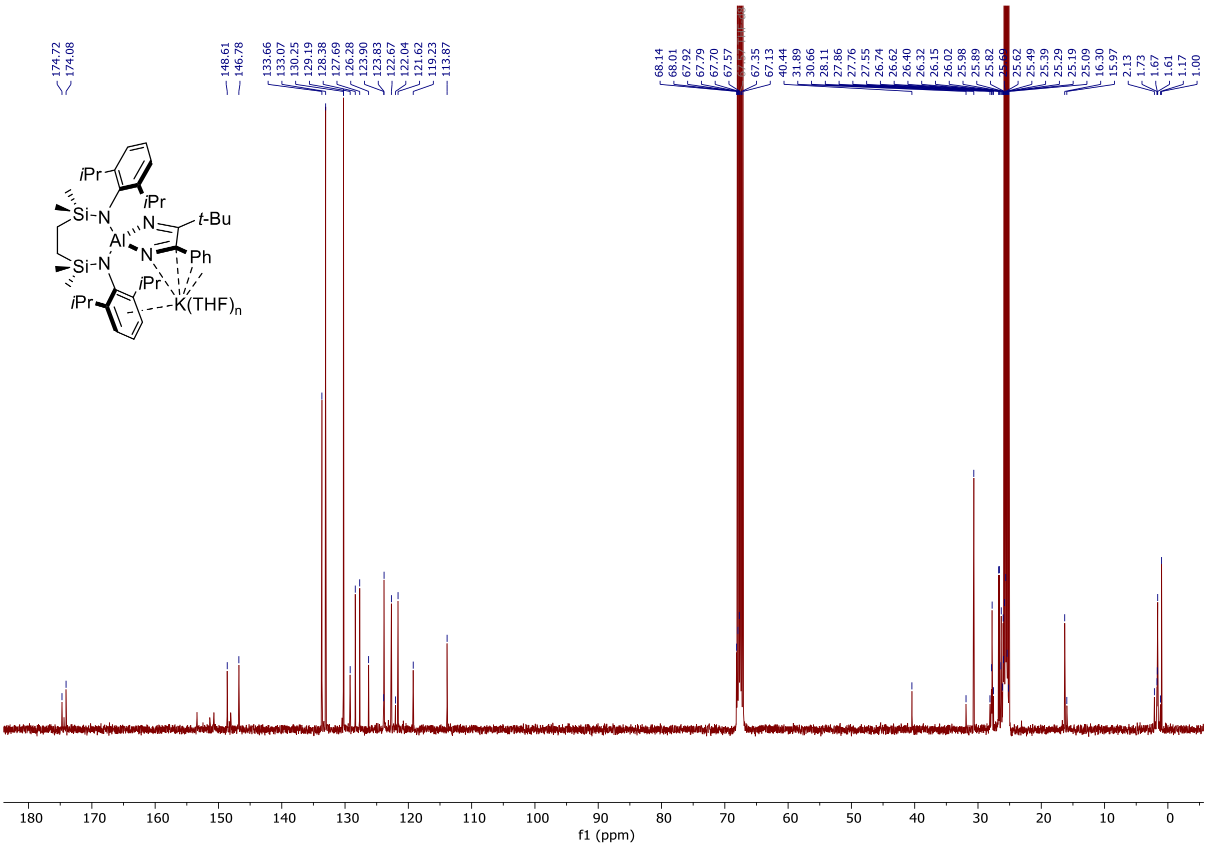

Figure S2*: $^{13}C\{^{1}H\}$ NMR (101 MHz, 298 K, d_8-THF) spectrum of Compound* ***8****.*

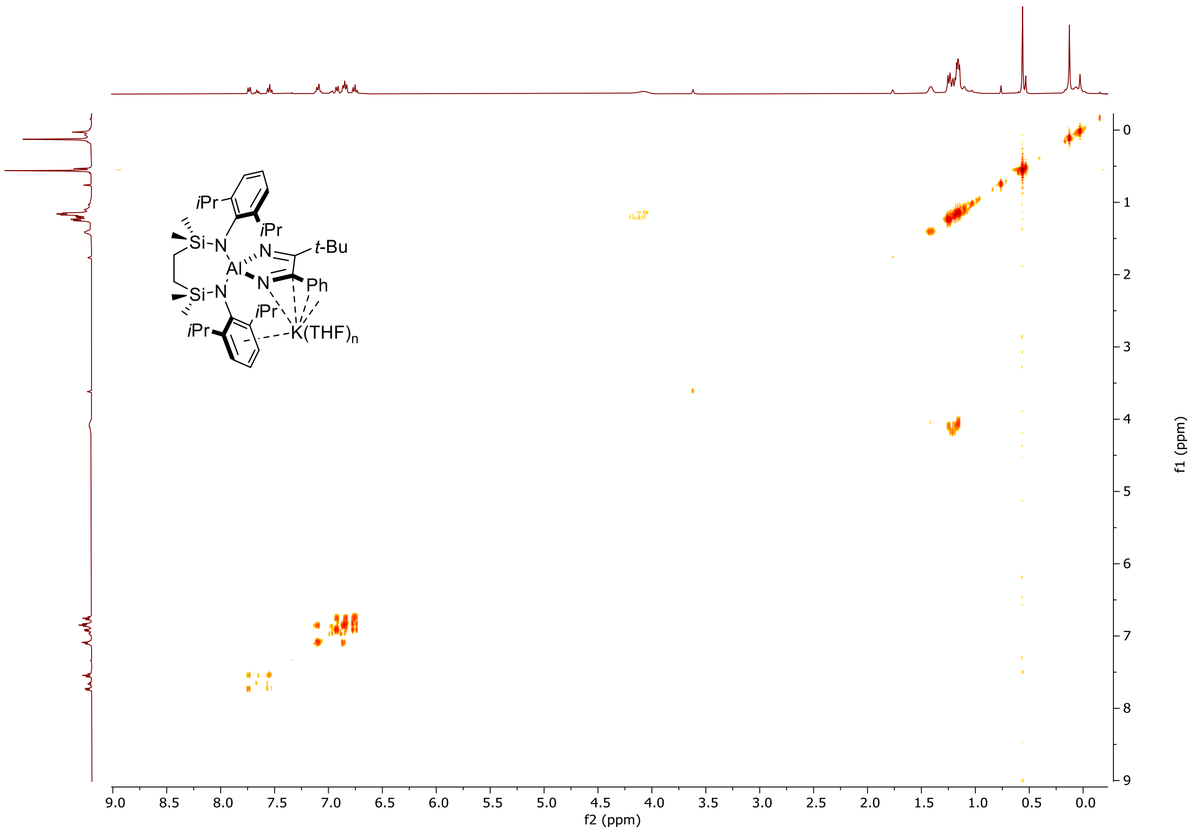

Figure S3. *¹H-¹H NMR COSY (298 K, d₈-THF) trace of* ***8***.

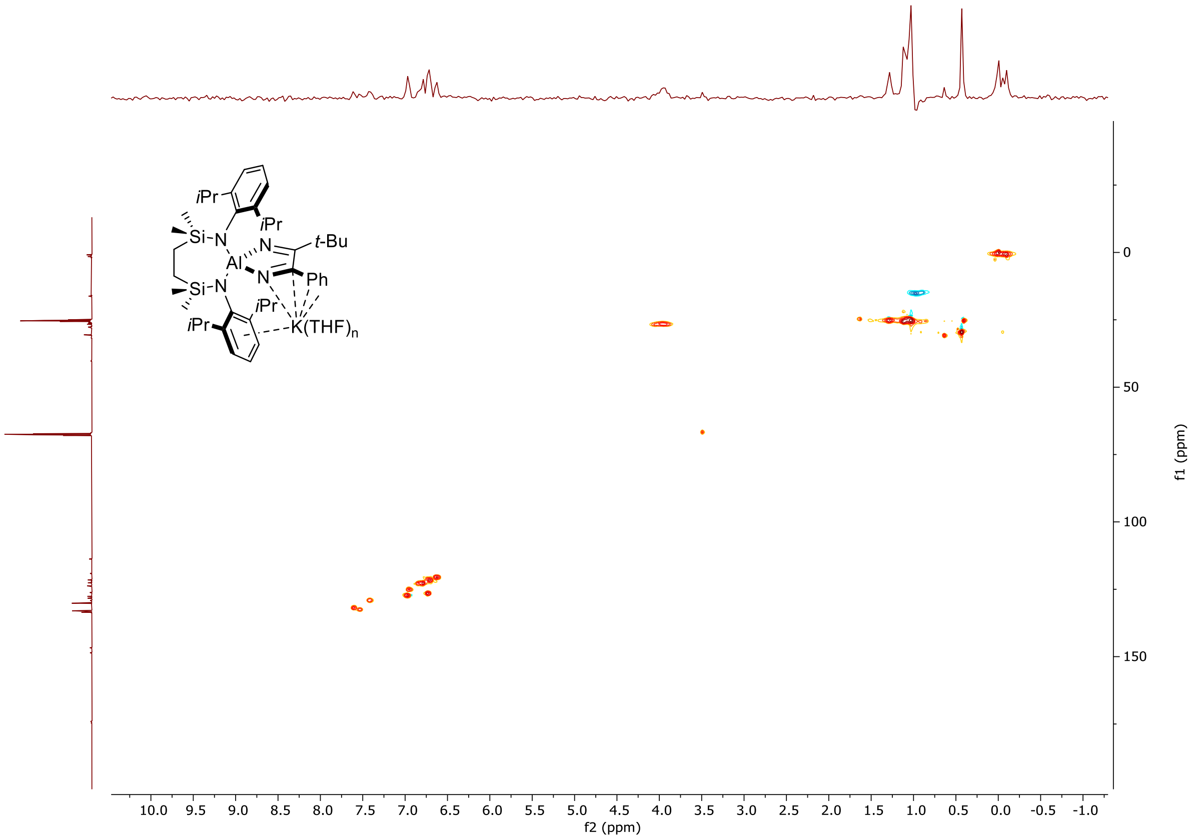

Figure S4. *¹H-¹³C NMR HSQC (298 K, d₈-THF) trace of* ***8***.

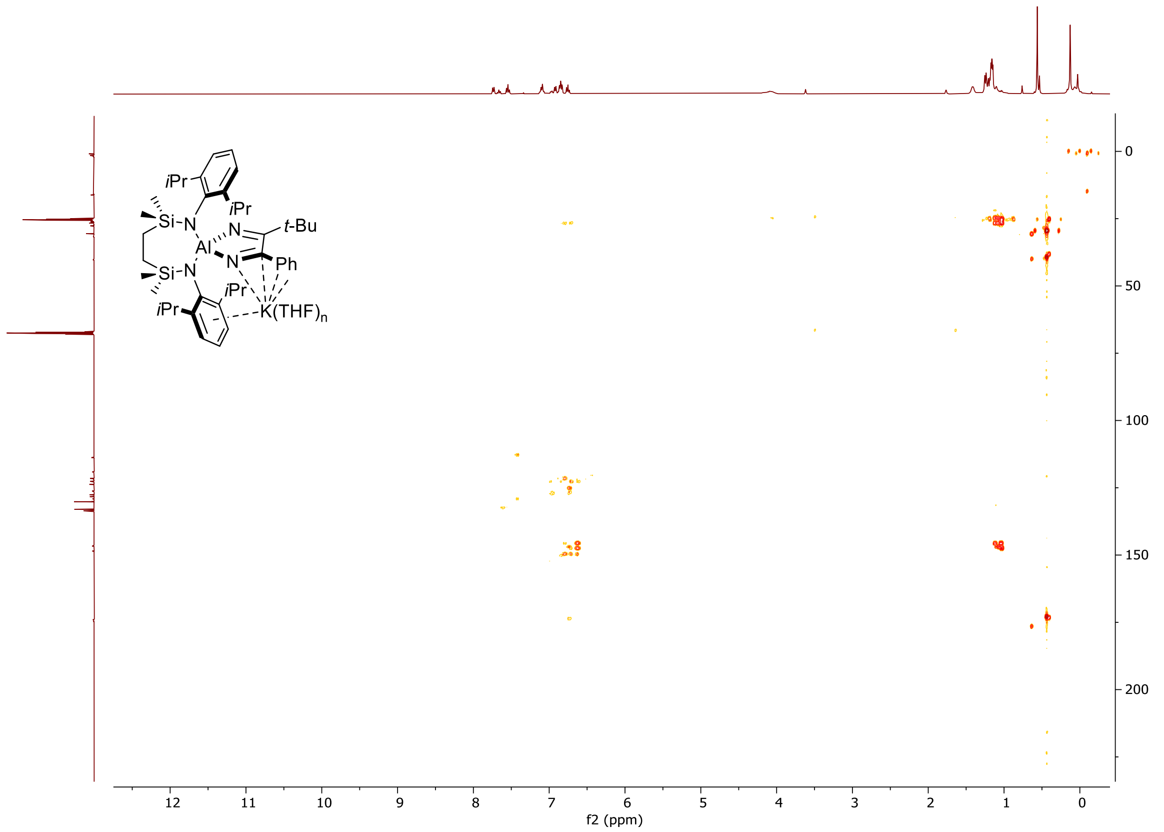

Figure S5. *^{1}H-^{13}C NMR HMBC (298 K, d_8-THF) trace of **8**.*

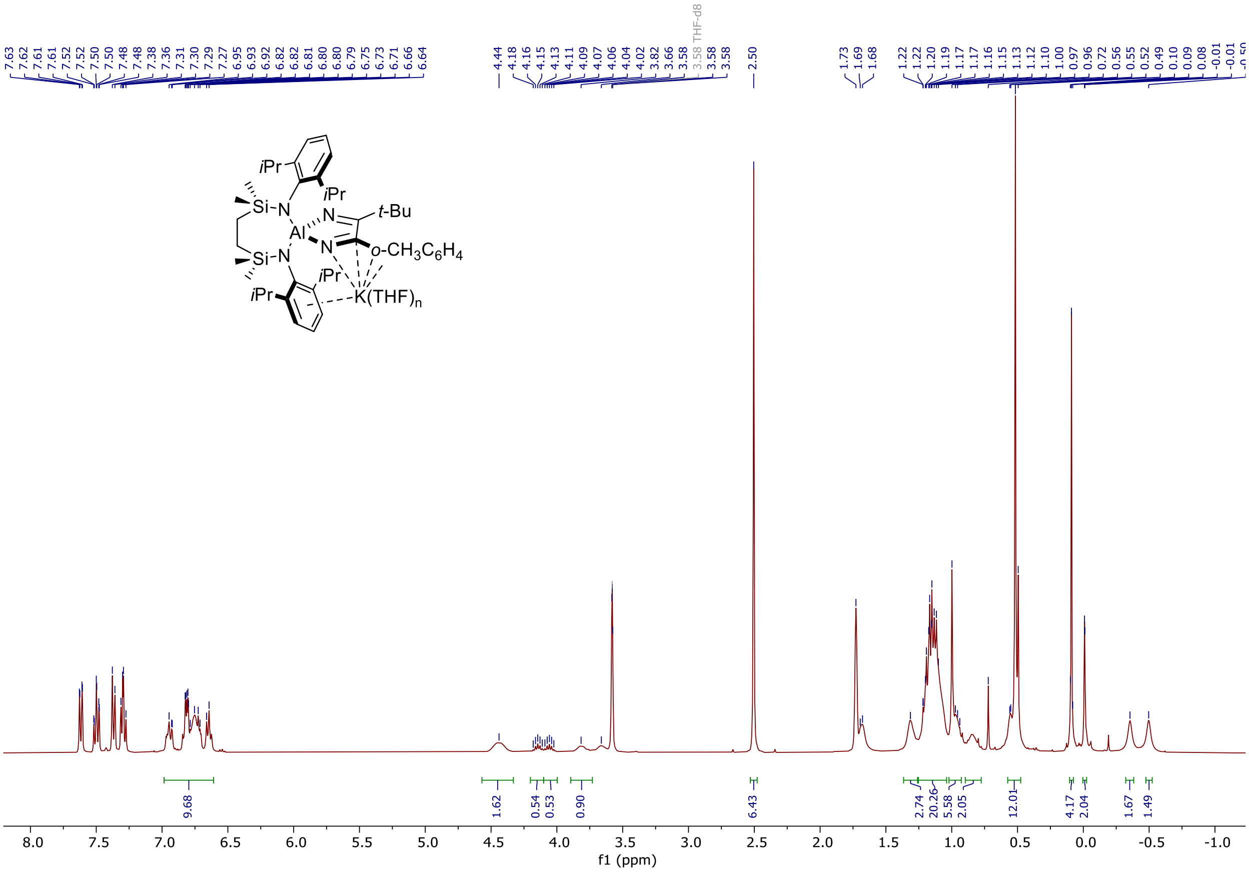

Figure S6: *^{1}H NMR (400 MHz, 298 K, d_8-THF) spectrum of Compound **9**.*

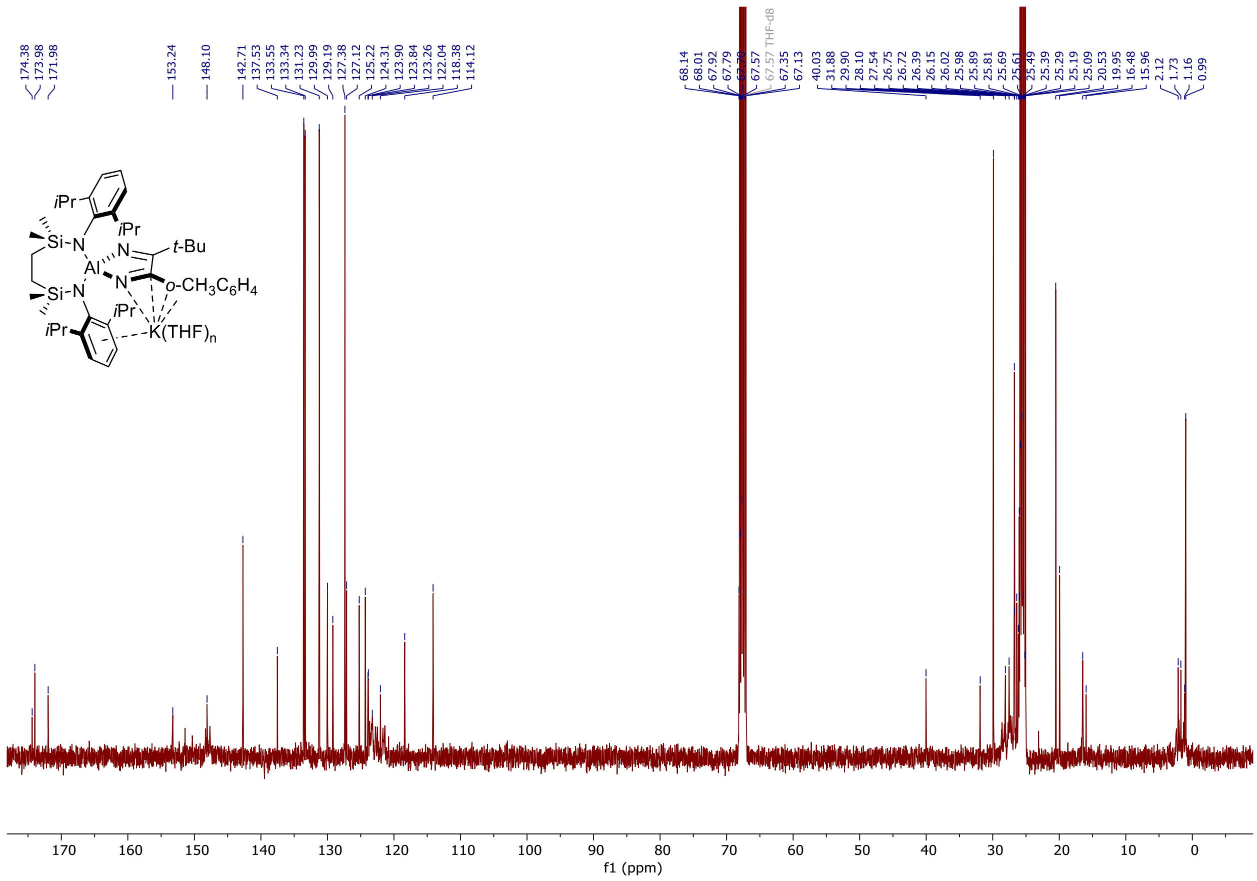

Figure S7: $^{13}C\{^1H\}$ *NMR (101 MHz, 298 K,* d_8*-THF) spectrum of Compound* ***9***

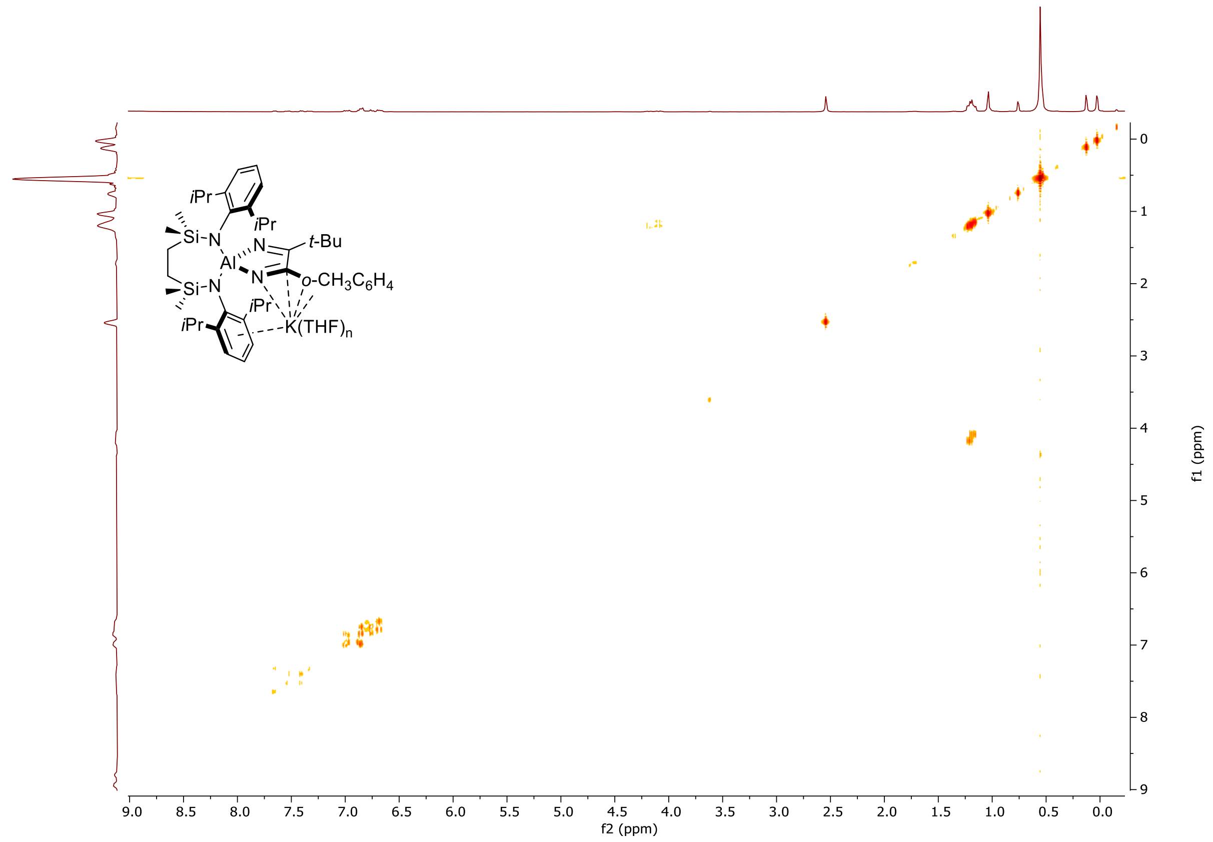

Figure S8. ^1H*-*^1H *NMR COSY (298 K,* d_8*-THF) trace of* ***9***.

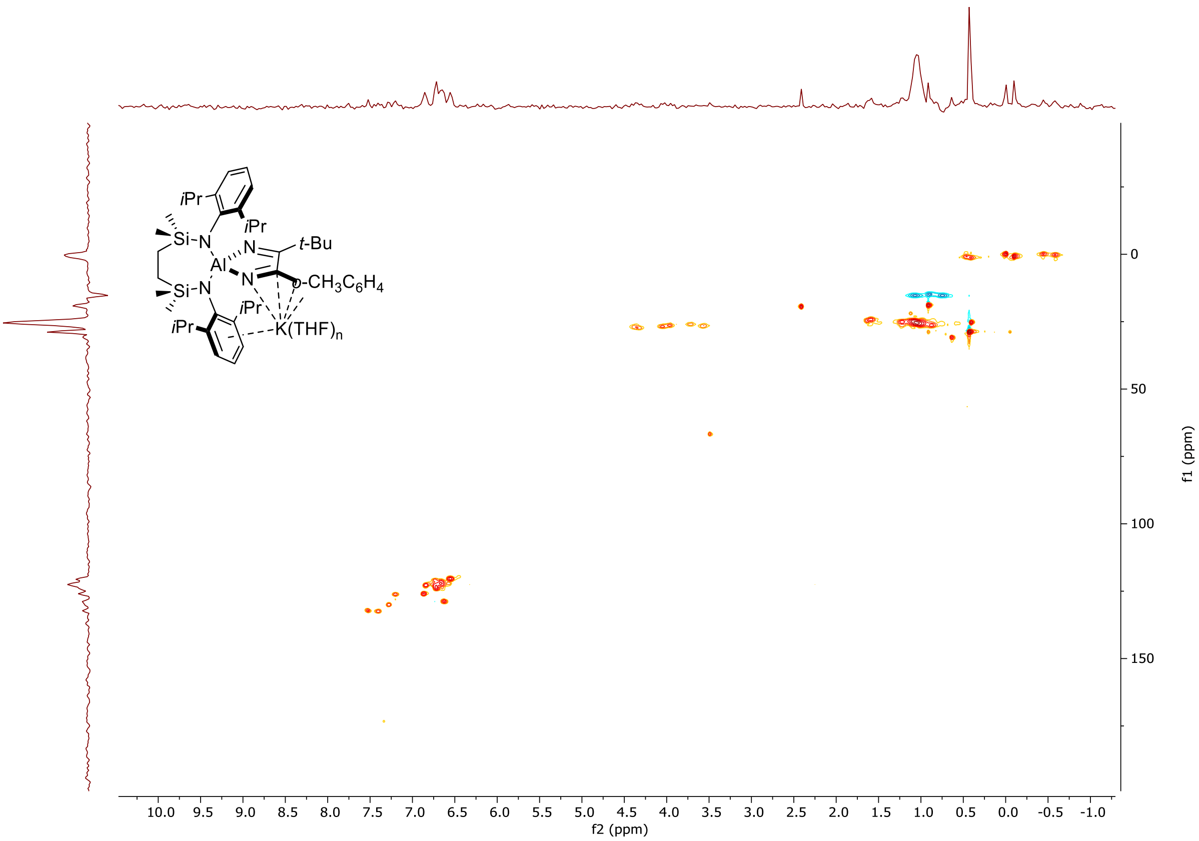

Figure S9. *^{1}H-^{13}C NMR HSQC (298 K, d₈-THF) trace of* ***9***.

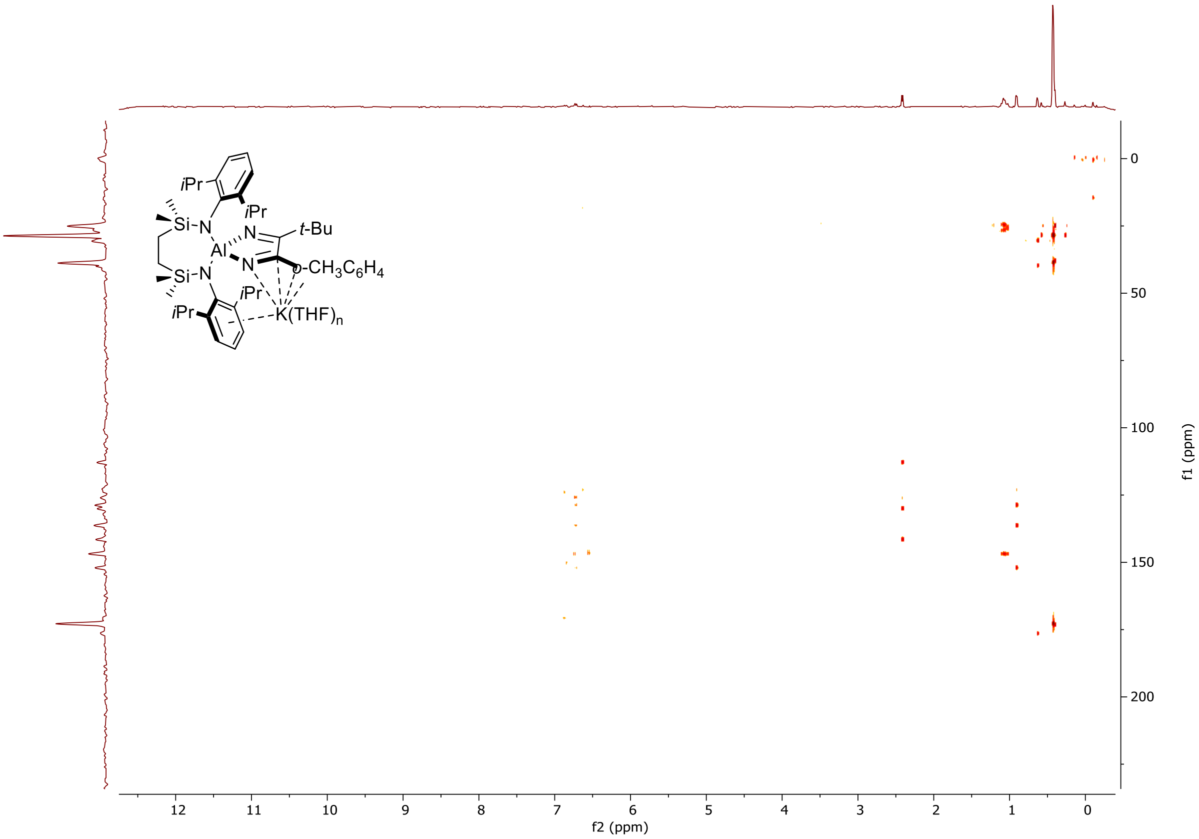

Figure S10. *^{1}H-^{13}C NMR HMBC (298 K, d₈-THF) trace of* ***9***.

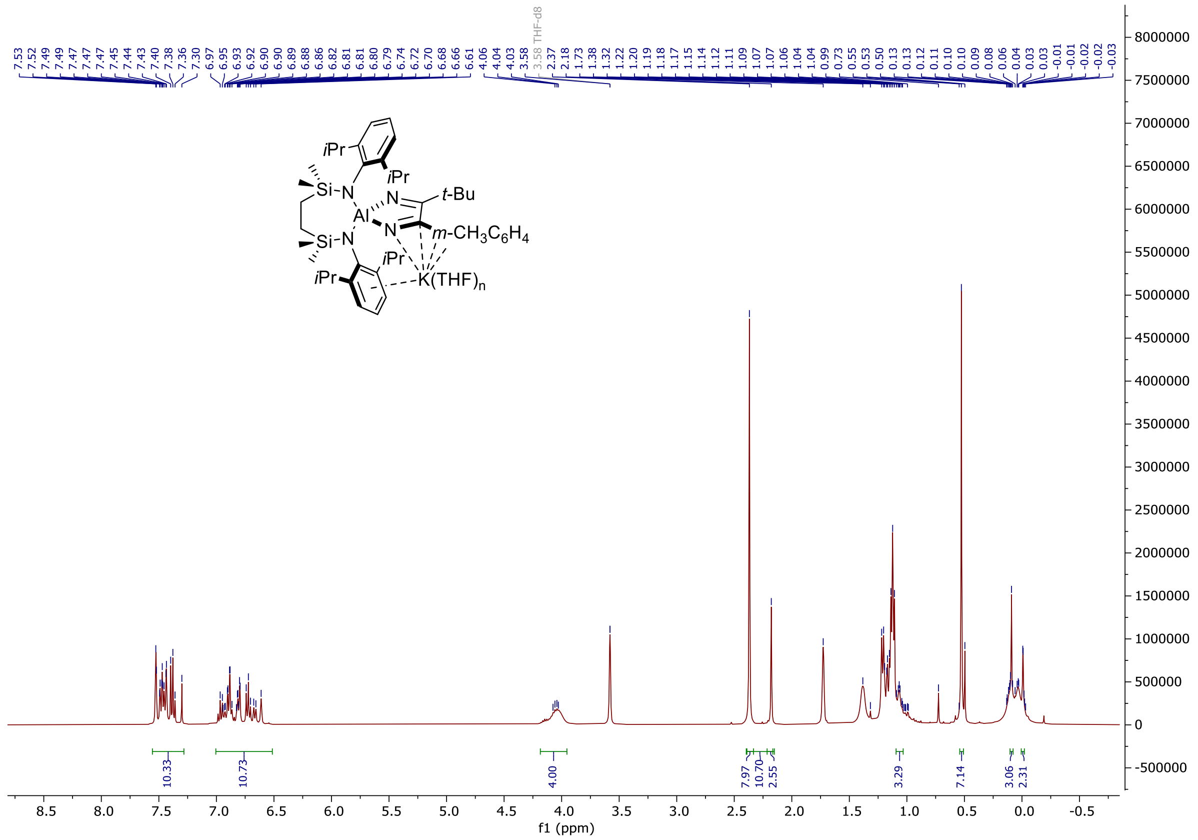

Figure S11: *^{1}H NMR (400 MHz, 298 K, d_8-THF) spectrum of Compound* ***10****.*

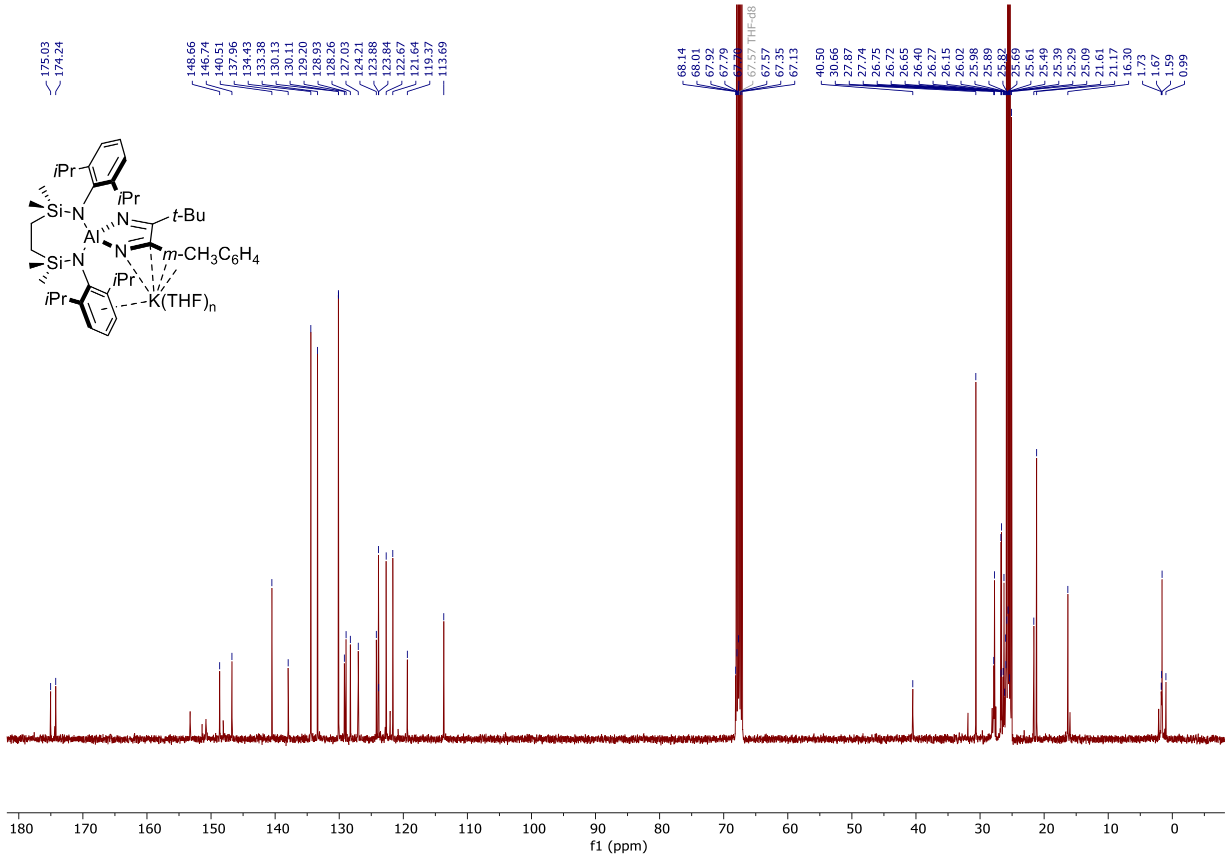

Figure S12*: ^{13}C{^{1}H} NMR (101 MHz, 298 K, d_8-THF) spectrum of Compound* ***10****.*

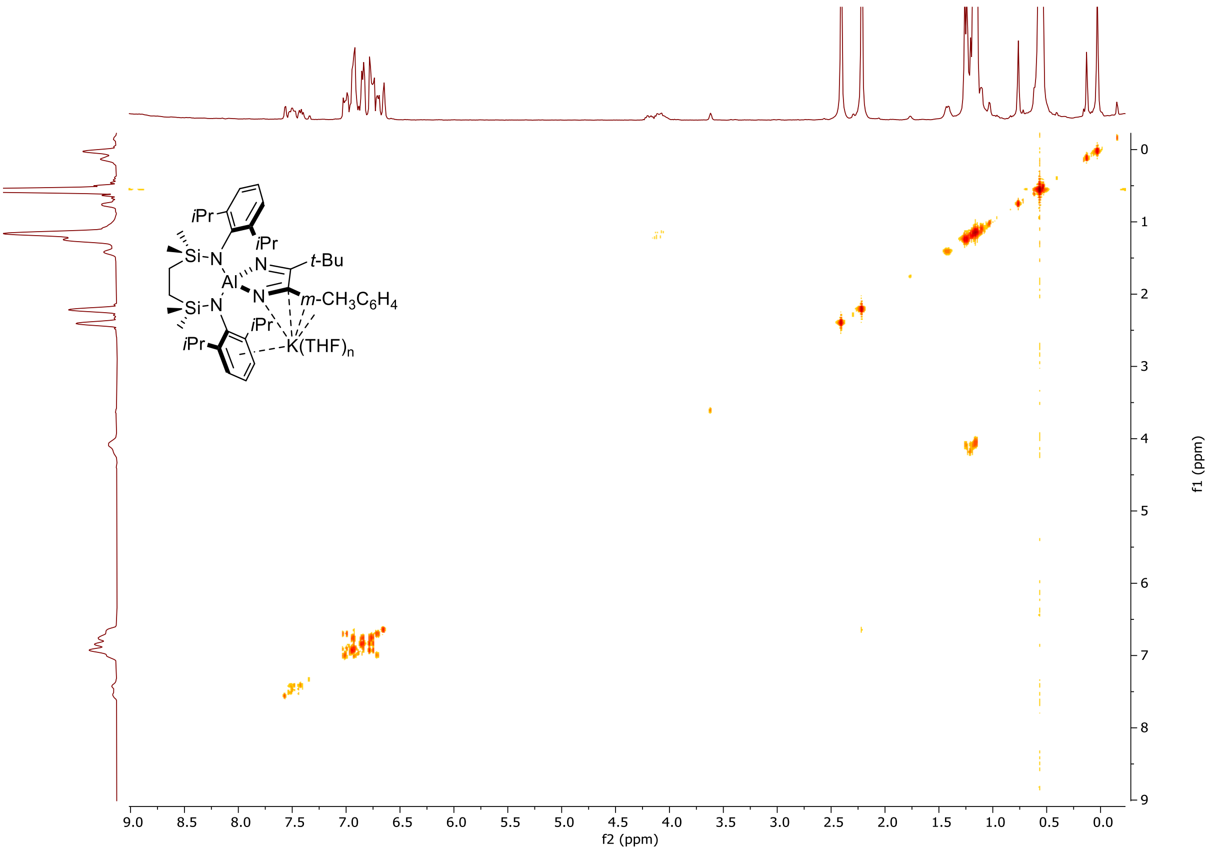

Figure S13. *^{1}H-^{1}H NMR COSY (298 K, d_8-THF) trace of **10**.*

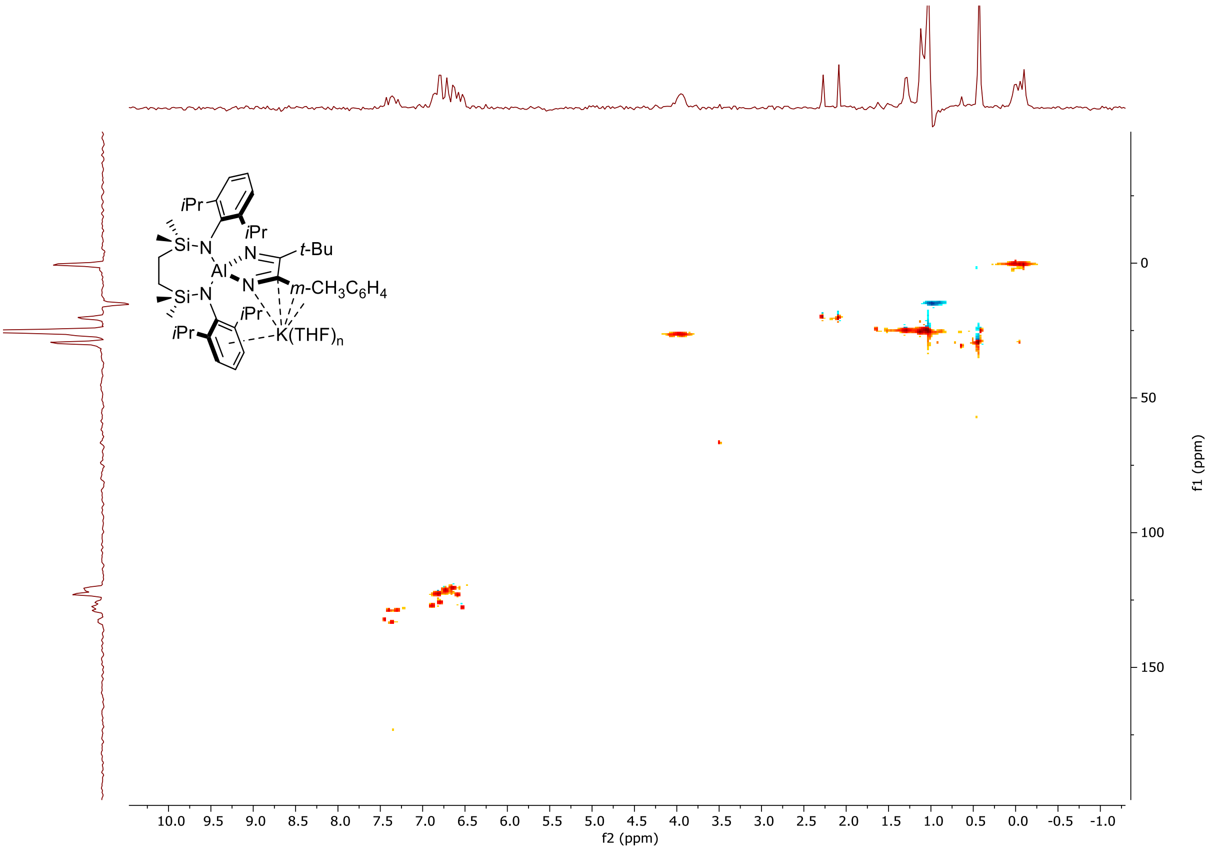

Figure S14. *^{1}H-^{13}C NMR HSQC (298 K, d_8-THF) trace of **10**.*

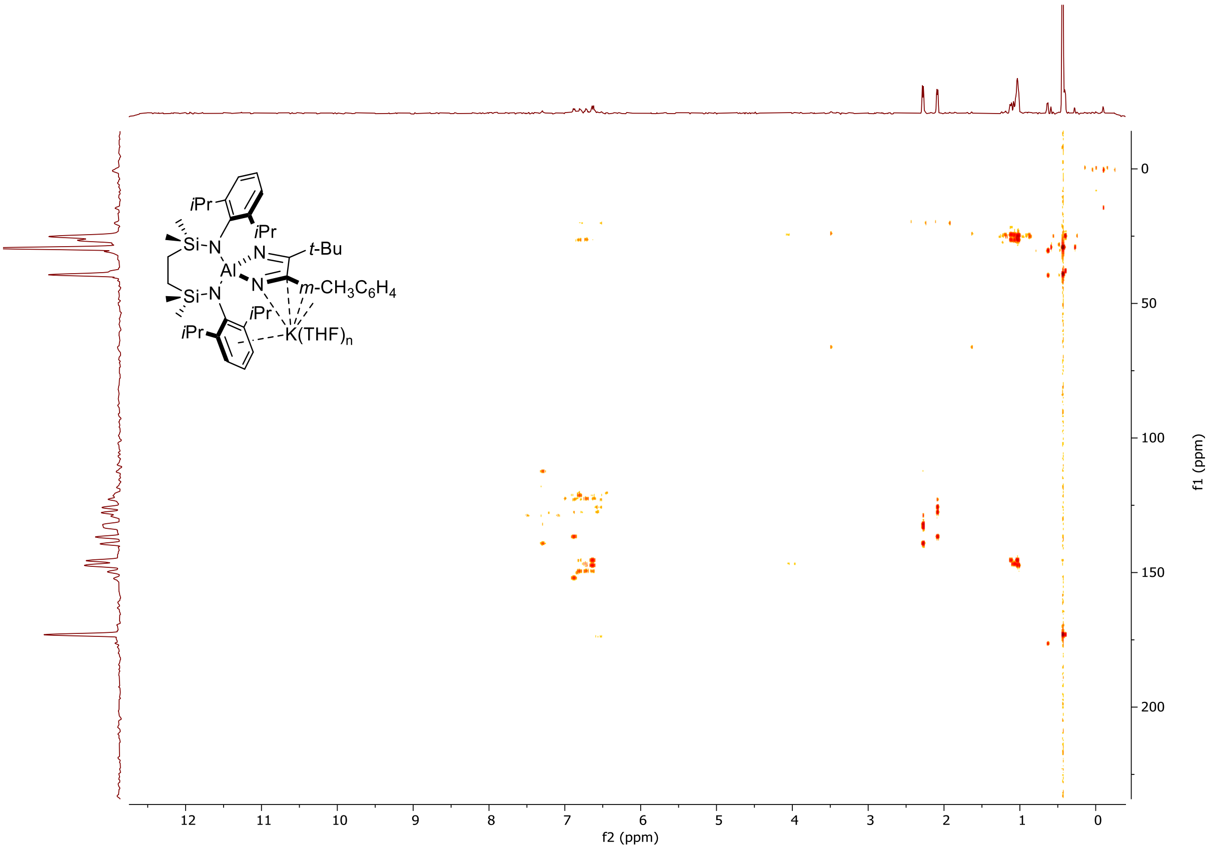

Figure S15. ^{1}H-^{13}C *NMR HMBC (298 K, d_8-THF) trace of* ***10***.

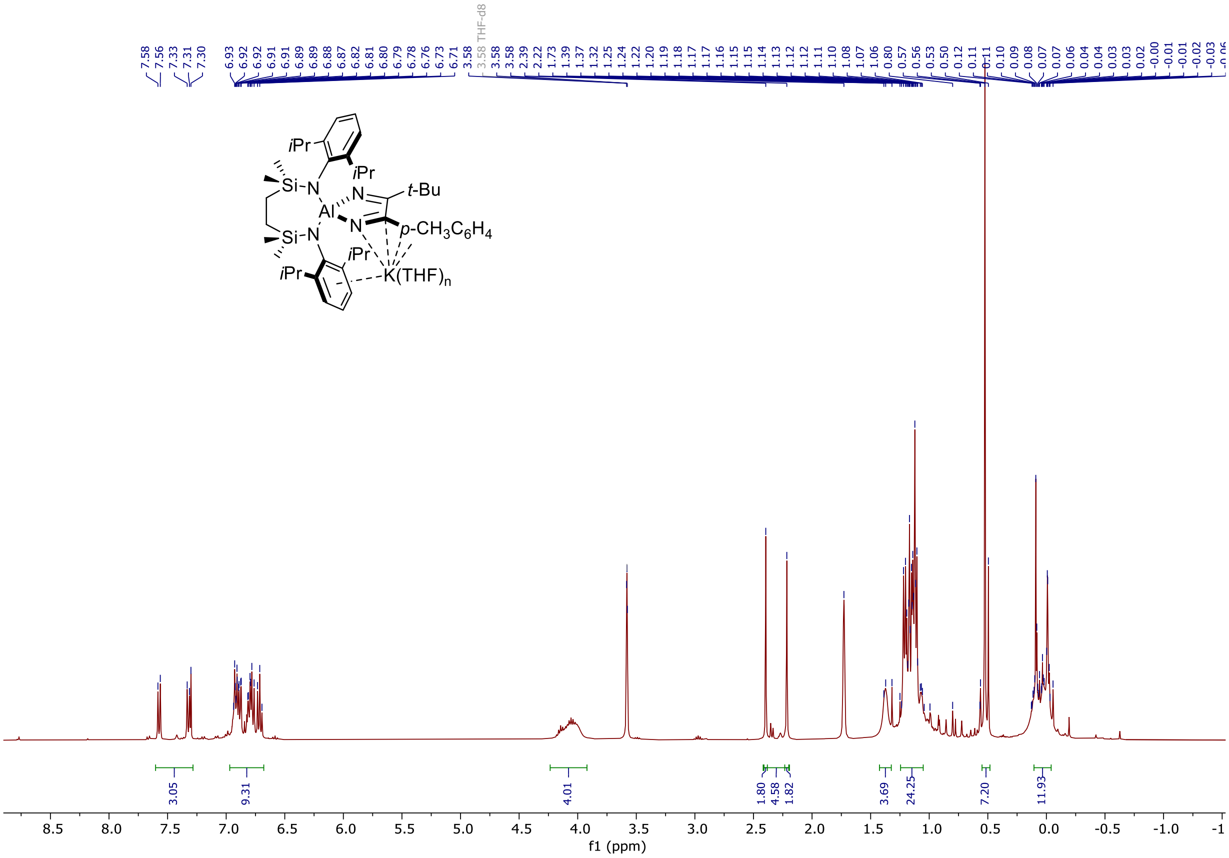

Figure S16: ^{1}H *NMR (400 MHz, 298 K, d_8-THF) spectrum of Compound* ***11***.

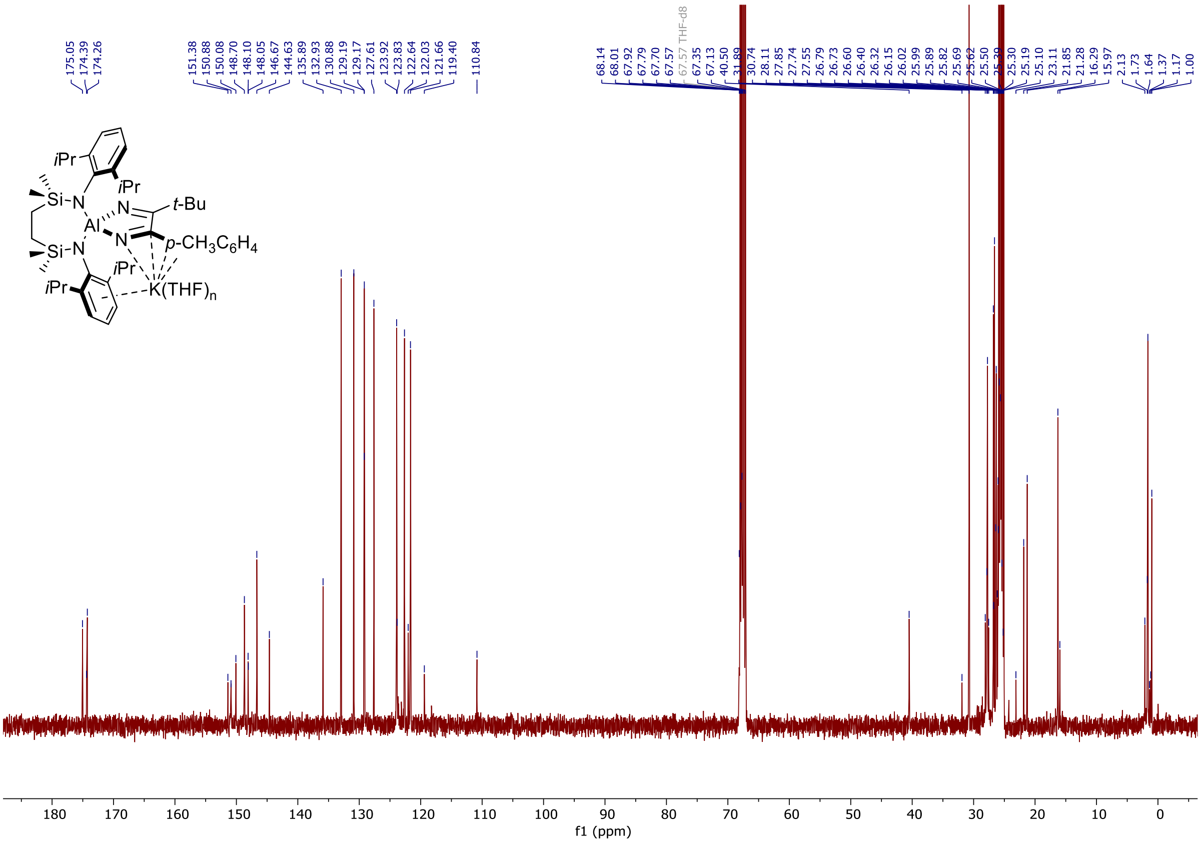

Figure S17: *$^{13}C\{^{1}H\}$ NMR (101 MHz, 298 K, d_8-THF) spectrum of Compound* ***11***.

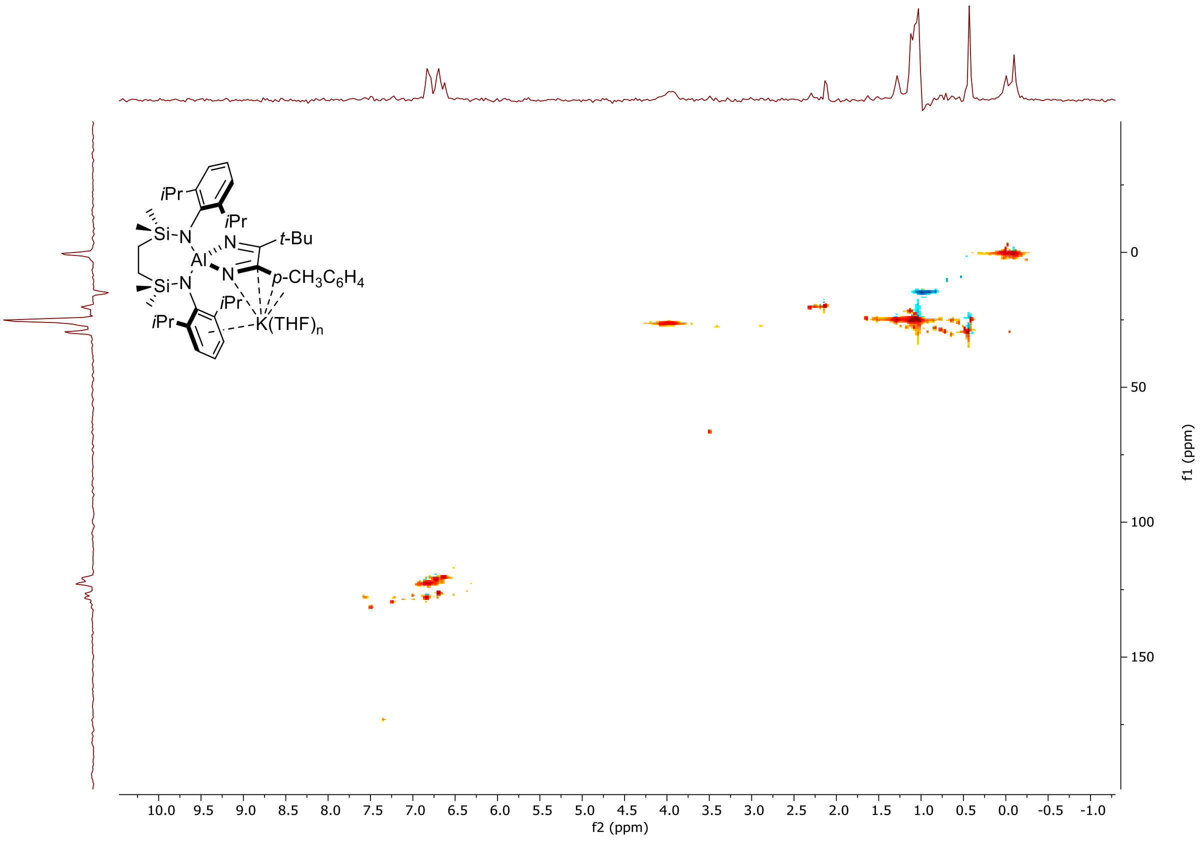

Figure S18. *^{1}H-^{13}C NMR HSQC (298 K, d_8-THF) trace of* ***11***.

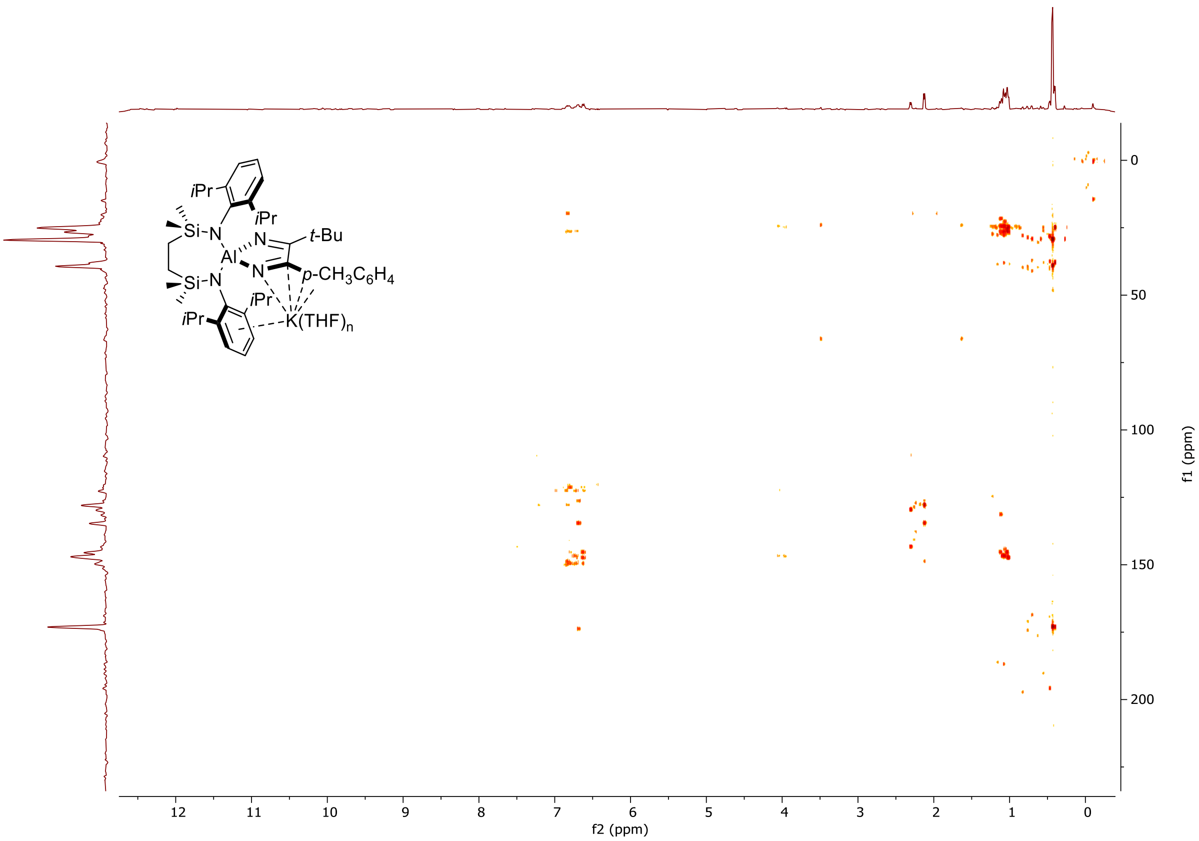

Figure S19. ^{1}H-^{13}C NMR HMBC (298 K, d_8-THF) trace of **11**.

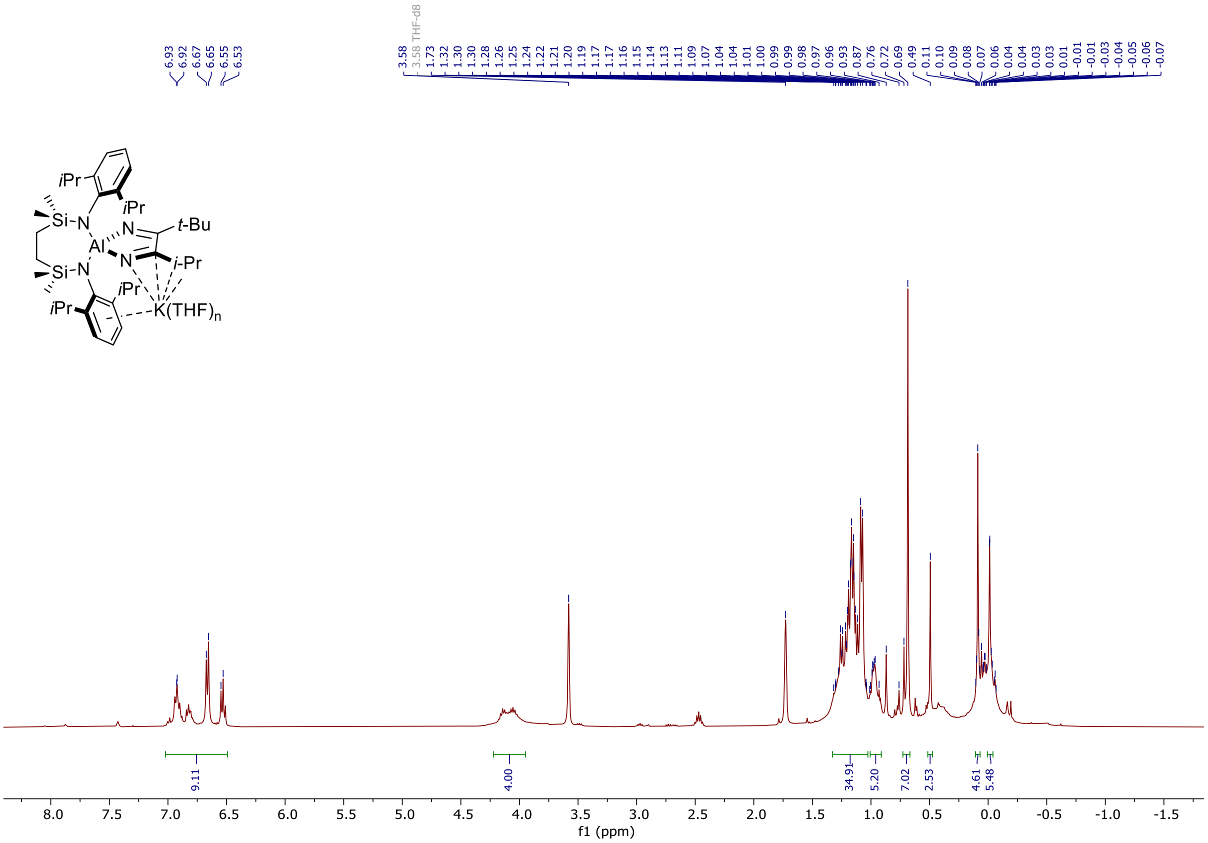

Figure S20: *^{1}H NMR (400 MHz, 298 K, d_8-THF) spectrum of Compound **12**.*

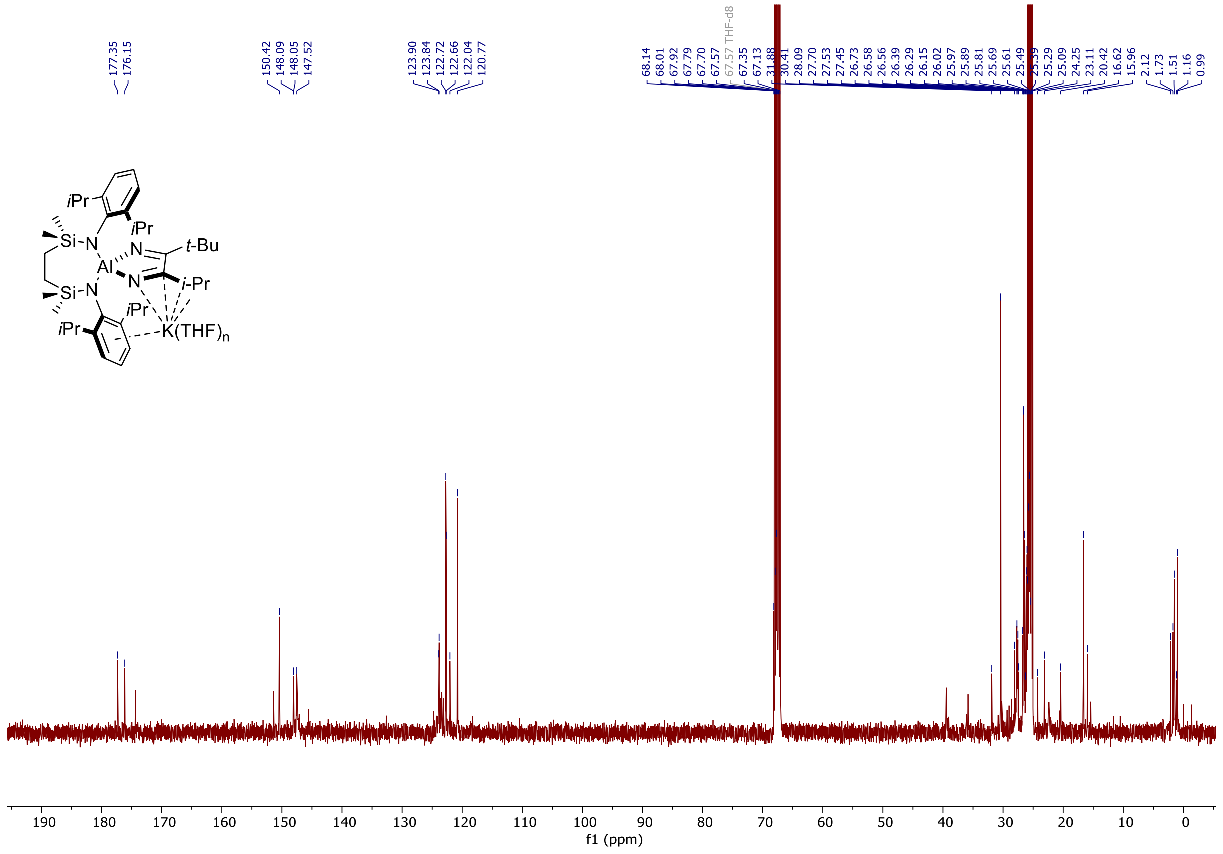

Figure S21: $^{13}C\{^{1}H\}$ *NMR (101 MHz, 298 K, d_8-THF) spectrum of Compound **12**.*

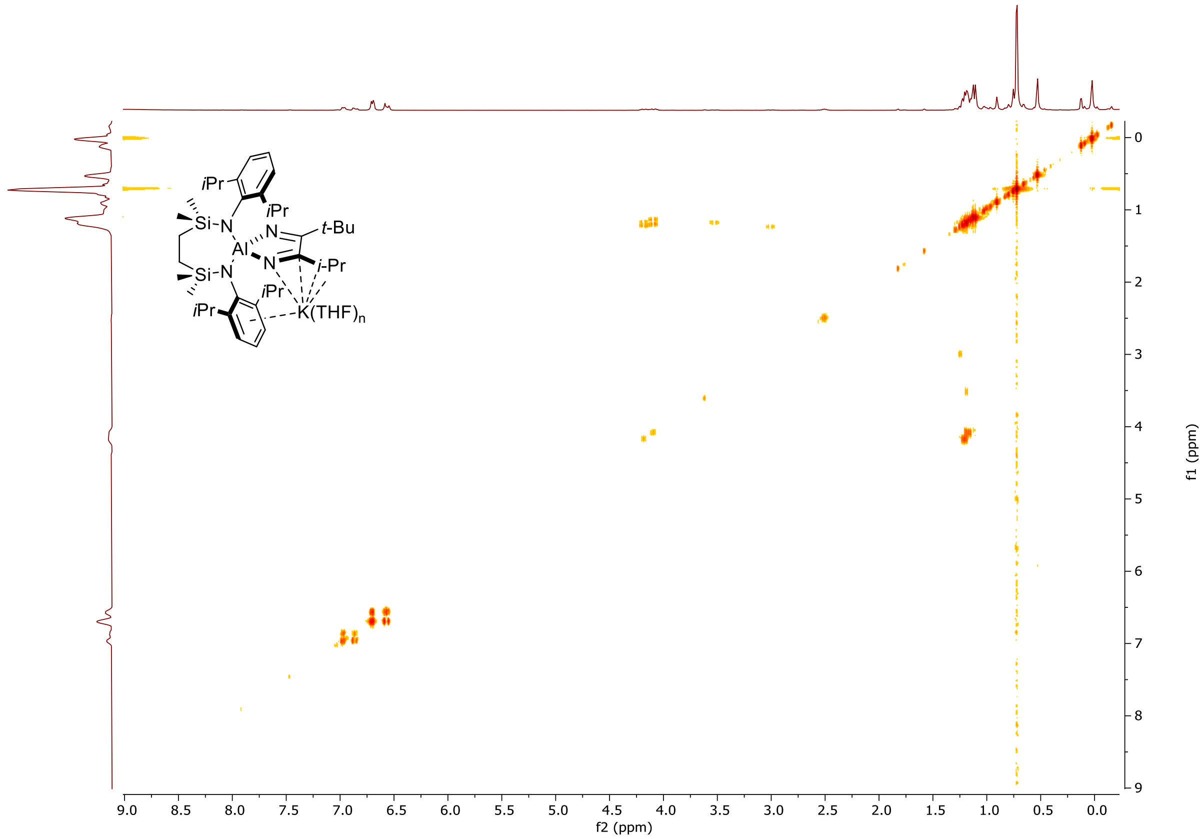

Figure S22. *^{1}H-^{1}H NMR COSY (298 K, d_8-THF) trace of **12**.*

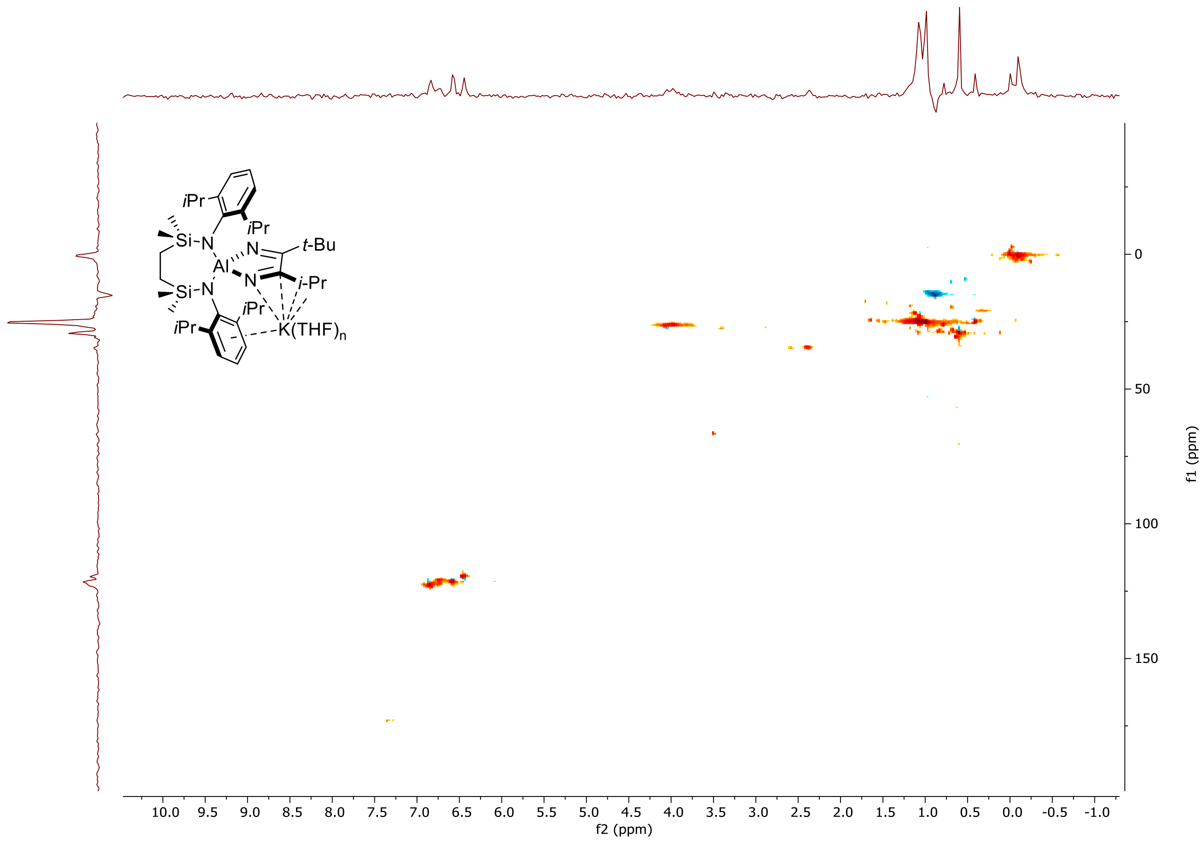

Figure S23. *^{1}H-^{13}C NMR HSQC (298 K, d_8-THF) trace of **12**.*

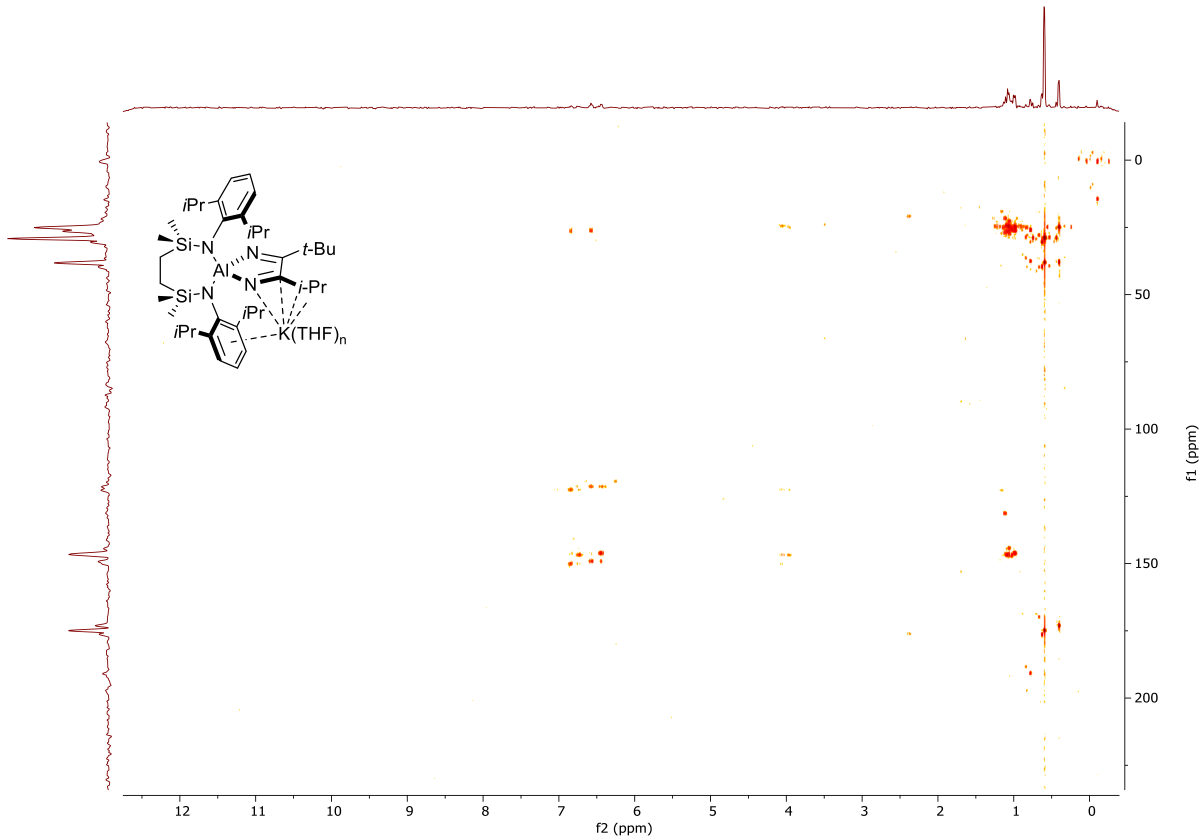

Figure S24. *^{1}H-^{13}C NMR HMBC (298 K, d_8-THF) trace of **12**.*

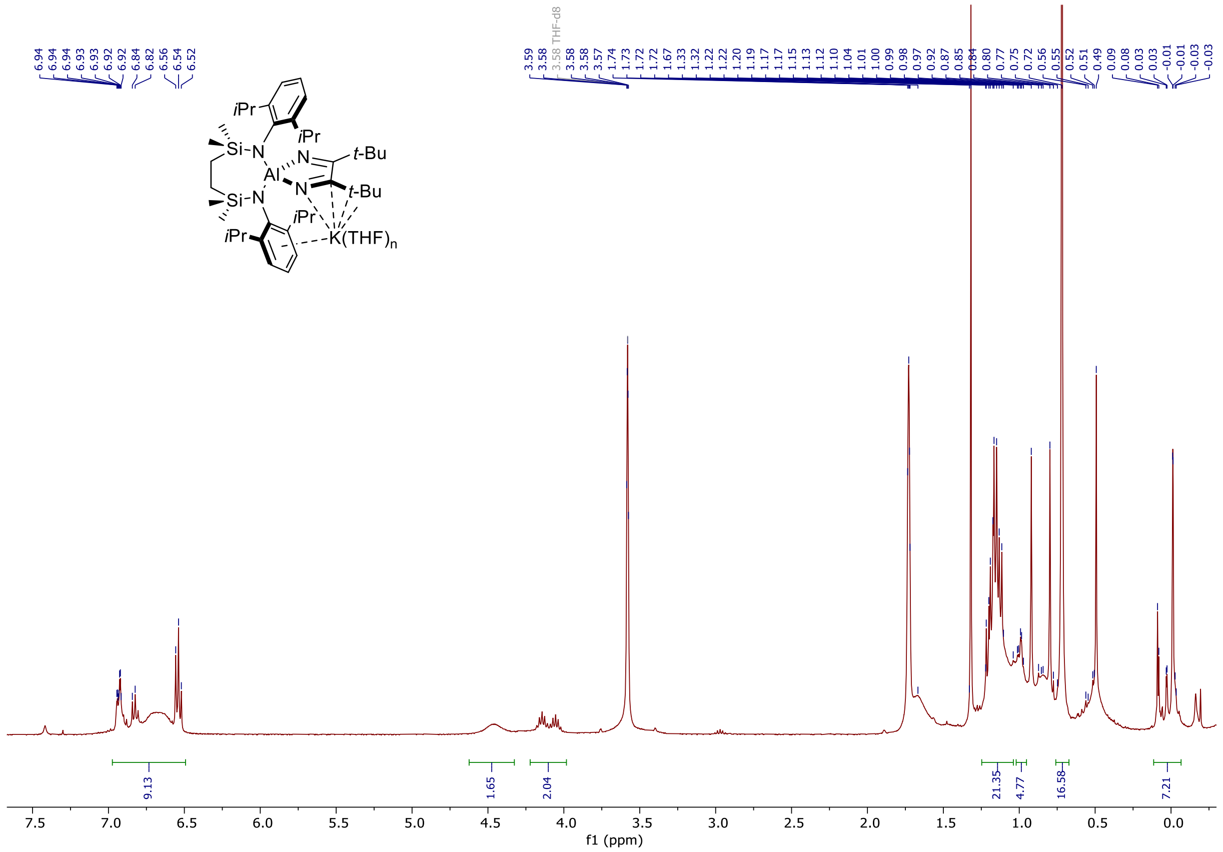

Figure S25: *^{1}H NMR (400 MHz, 298 K, d_8-THF) spectrum of Compound* ***13****.*

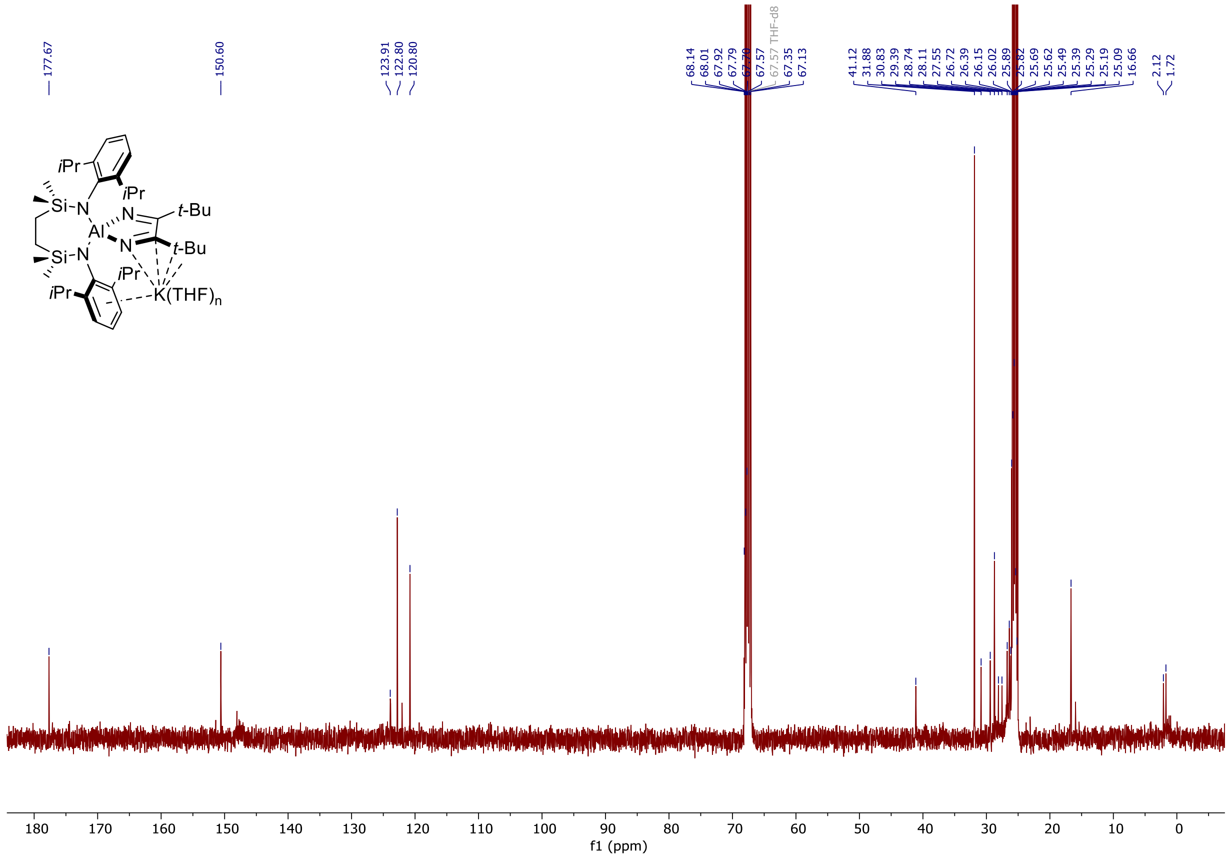

Figure S26*: ^{13}C{^{1}H} NMR (101 MHz, 298 K, d_8-THF) spectrum of Compound* ***13****.*

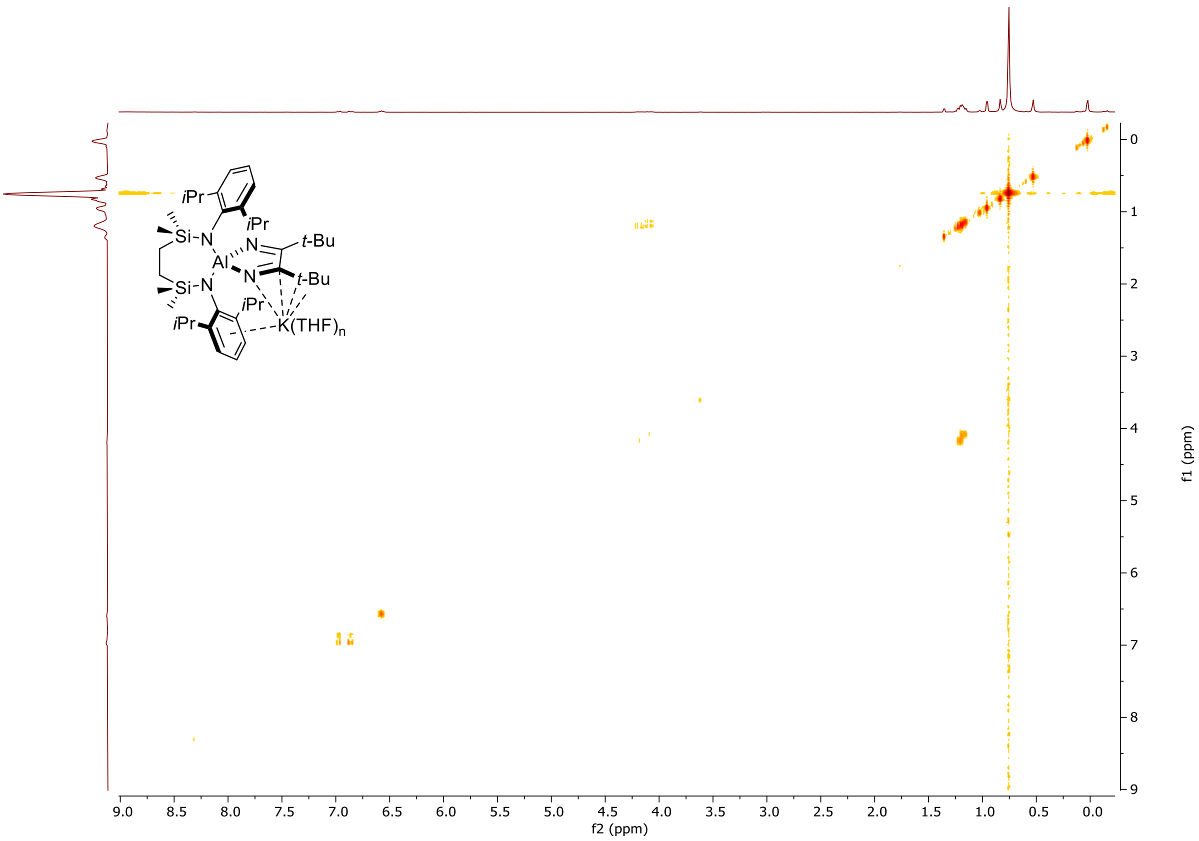

Figure S27. *^{1}H-^{1}H NMR COSY (298 K, d_8-THF) trace of* ***13***.

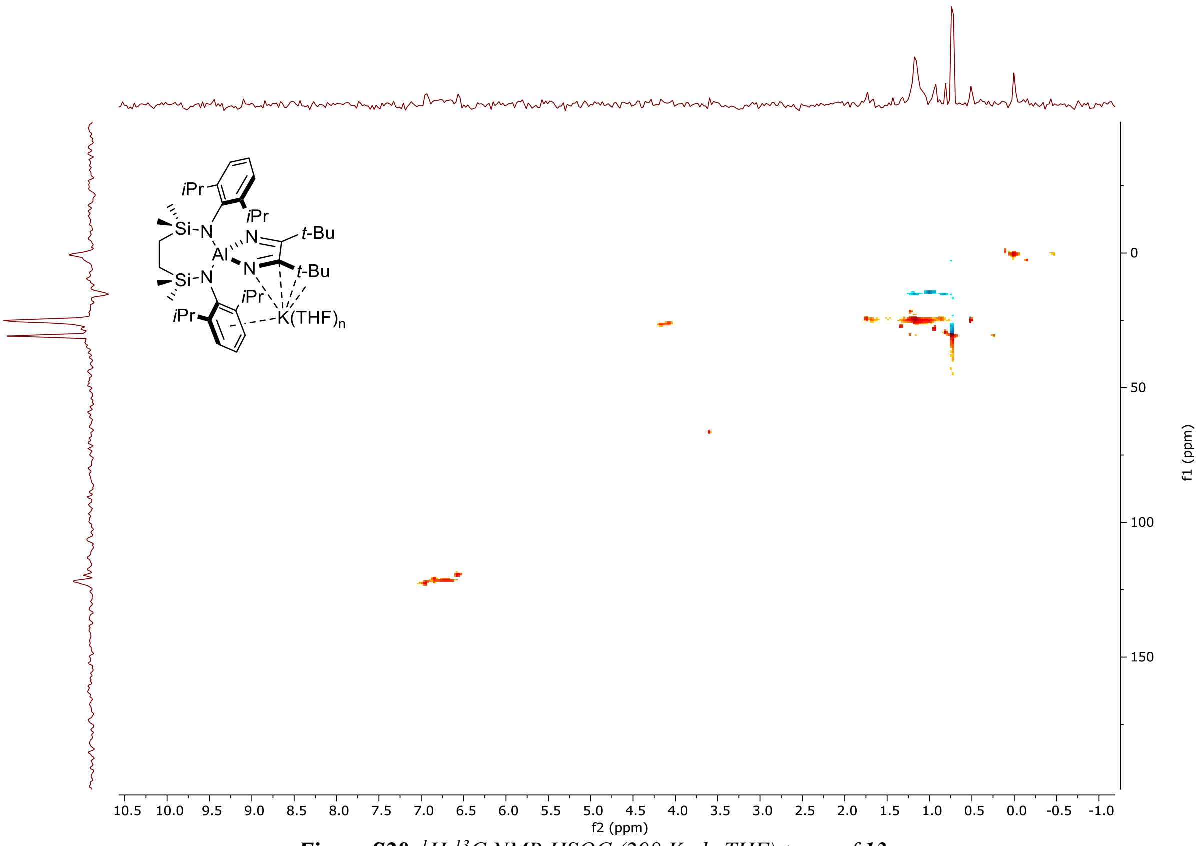

Figure S28. *^{1}H-^{13}C NMR HSQC (298 K, d_8-THF) trace of* ***13***.

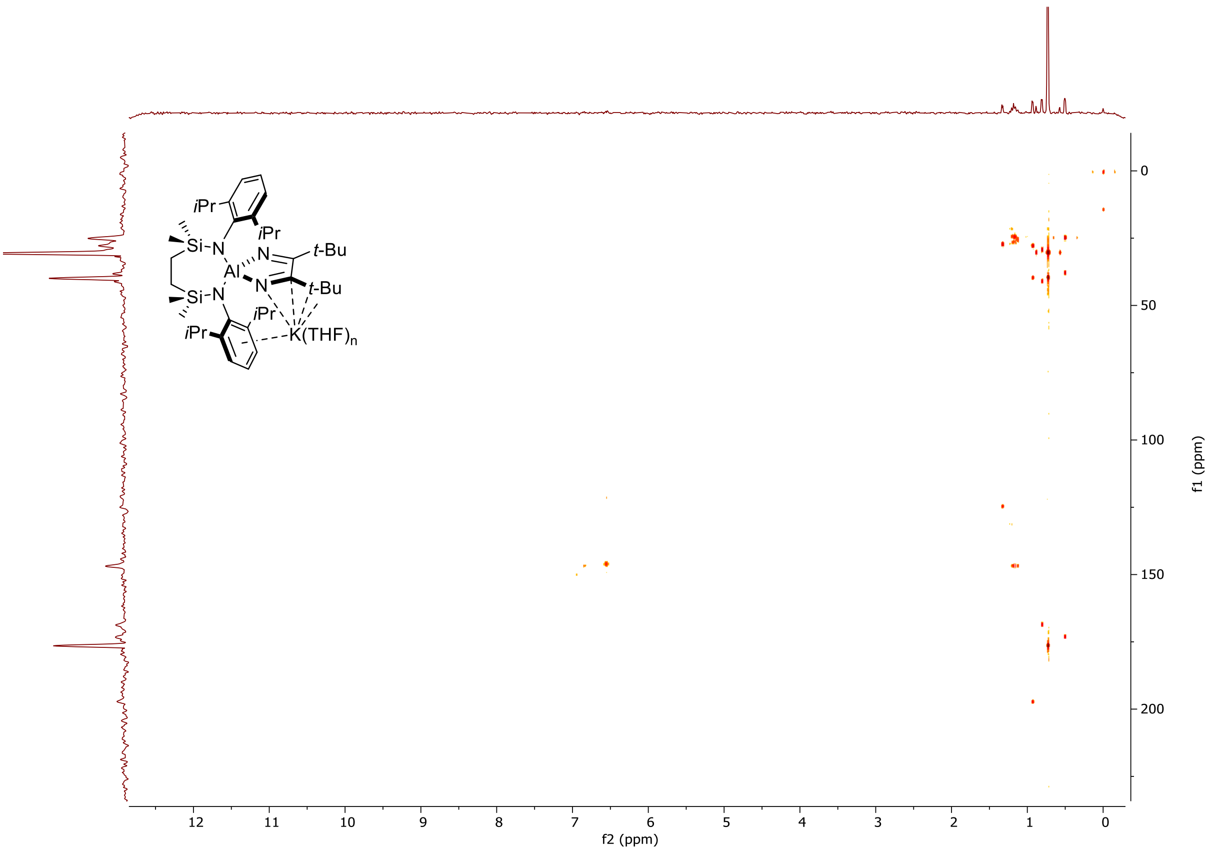

Figure S29. *^{1}H-^{13}C NMR HMBC (298 K, d_8-THF) trace of **13**.*

X-ray Crystallography

Single Crystal X-ray diffraction data for compounds **12** and **13** were collected on an Agilent SuperNova EosS2 diffractometer using Cu-Kα (1.54184 Å) radiation, whilst compounds **9** and **10** were collected on XtaLAB Synergy, Dualflex, HyPix-Arc 100 diffractometer Cu-Kα (1.54184 Å) radiation. In each case, the crystals were maintained at 150 K during data collection. Using Olex2,[2] the structures were solved with the olex2.solve[3] structure solution program or ShelXT and refined with the ShelXL[4] refinement package using Least−Squares minimisation.

Due to substantial drop-off in diffraction intensity at high angles, a resolution limit of 0.82 Å was applied to the data of compound **9**. The crystal structure contains one aluminium-potassium complex and one region of disordered solvent per asymmetric unit. The latter moiety was discerned to be a molecule of *n*-hexane disordered over at least three components. Attempts to model this disorder were unsatisfactory. Instead, a solvent mask was calculated, and 216 electrons were found in a volume of 1196 $Å^3$ in 2 voids per unit cell. This is consistent with the presence of 1 n-hexane molecule per Asymmetric Unit which account for 200 electrons per unit cell. In the metal complex, one of the di-isopropyl-phenyl groups was disordered and modelled in two components of relative occupancy 0.752(17):0.248(17). Further, each of the three potassium-bound THF-moieties was significantly disordered; the O1/O1A-containing THF moiety was modelled in two components of occupancy 0.604(8):0.396(8); the CH_2 groups of the O2-containing THF moiety were modelled in two components of occupancy 0.589(14):0.412(14) with O2 in part 0; the CH_2 groups of the O3-containing THF moiety were modelled in two components of occupancy 0.770(6):0.230(6) with O3 in part 0.

The asymmetric unit of **10** comprises of one aluminum-based molecule with an incorporated potassium atom and two coordinated THF molecules. Two-fold disorder was identified and readily modelled on the THF molecules, (O1,C44-C47; O2,C48-C51), and *m*-tolyl (C37-C43), N=C-C=N (N3=C31-C32=N4) units, in a 60:40, 60:30 and 70:30 ratio, respectively. Appropriate distance and ADP restraints were employed to assist convergence.

The asymmetric unit of **12** is comprises of one aluminum-based molecule with an incorporated potassium atom and three coordinated THF molecules. Two-fold disorder was identified and readily modelled on the three THF units (O1,C40-C43; O2,C44-C47; O3,C48-C51) in a 70:30, 50:50 and 60:40 ratio, respectively. Appropriate distance and ADP restraints were employed to assist convergence.

The asymmetric unit in **13** is composed of two molecules with three thf units incorporated in each molecule. Disorder was identified and readily modelled on each thf unit (O1,41-44 (50:50 ratio); O2, 45-48 (60:40 ratio); O3, 49-52 (45:55 ratio); O4, 93-96 (50:50 ratio); O5, 97-100 (75:25 ratio); O6, 101-104 (45:55 ratio)). Furthermore, two-fold disorder was located on one of the $\{Si(CH_3)NCH_2\}_2$ (Si3, Si4, C53-58) backbones as well as all four of the tert-butyl fragments (C89-92; C85-88; C33-36; C37-40), readily modelled in a 70:30 and 90:10 ratio, respectively. Appropriate distance and ADP restraints were implemented to assist convergence.

Table S2. Crystal Data and Structure Refinement for Compounds **9**, **10**, **12** and **13**.

Identification code	y25msh49 (**9**)	y25msh47 (**10**)	s25msh34 (**12**)	s25msh36 (**13**)
Empirical formula	$C_{61}H_{103}AlKN_4O_3Si_2$	$C_{51}H_{81}AlKN_4O_2Si_2$	$C_{51}H_{90}AlKN_4O_3Si_2$	$C_{52}H_{92}AlKN_4O_3Si_2$
Formula weight	1062.73	904.45	929.52	943.55
Crystal system	monoclinic	orthorhombic	monoclinic	monoclinic
Space group	$P2_1/c$	*Pbca*	$P2_1/c$	$P2_1/c$
a/ Å	11.9157(4)	21.72420(10)	18.7855(5)	23.34750(10)
b/ Å	22.0856(7)	21.23830(10)	13.8683(4)	18.77060(10)
c/ Å	24.5623(8)	22.94810(10)	21.3516(6)	27.9768(2)
α/ °	90	90	90	90
β/ °	90.479(3)	90	92.027(2)	113.6580(10)
γ/ °	90	90	90	90
U/ Å^3	6463.7(4)	10587.91(8)	5559.1(3)	11230.30(14)
Z	4	8	4	8
ρ_{calc}/ g cm^{-3}	1.092	1.135	1.111	1.116
μ/ mm^{-1}	1.529	1.775	1.713	1.702
F(000)	2324.0	3928.0	2032.0	4128.0
Crystal size/ mm^3	0.55 × 0.07 × 0.05	0.5 × 0.31 × 0.19	0.1 × 0.07 × 0.06	0.3 × 0.24 × 0.16
2θ range for data collection/°	5.382 to 140.15	5.602 to 160.41	7.602 to 147.006	6.898 to 146.556
Index ranges	$-14 \le h \le 14$	$-27 \le h \le 27$	$-16 \le h \le 23$	$-28 \le h \le 28$
	$-26 \le k \le 26$	$-26 \le k \le 26$	$-17 \le k \le 16$	$-23 \le k \le 16$
	$-29 \le l \le 29$	$-29 \le l \le 25$	$-26 \le l \le 26$	$-34 \le l \le 34$
Reflections collected	82749	169715	41316	155599
Independent reflections, R_{int}	12282, 0.1087	11769, 0.0362	11041, 0.0685	22469, 0.0330
Data/restraints/parameters	12282/747/850	11769/938/744	11041/214/715	22469/362/1665
Goodness–of–fit on F^2	1.081	1.044	1.070	1.013
Final R1, wR2 [I>=2σ(I)]	0.0901, 0.2756	0.0904, 0.2554	0.0750, 0.1927	0.0364, 0.1015
Final R1, wR2 [all data]	0.1126, 0.2980	0.0948, 0.2597	0.1303, 0.2506	0.0393, 0.1043
Largest diff. peak/hole/ e Å^{-3}	0.48/-0.32	1.59/-0.92	0.41/-0.66	0.34/-0.29

References

1. Shere, H. T. W.; Liu, H.-Y.; Hill, M. S.; Mahon, M. F., Alumanyl Reduction, Reductive Coupling and C–H Isomerization of Organic Nitriles. *Organometallics* **2024,** *43* (17), 1938-1945.
2. Dolomanov, O.V.; Bourhis, L. J.; Gildea, R. J.; Howard, J. A. K.; Puschmann, H. OLEX2: a complete structure solution, refinement and analysis program. *J. Appl. Cryst.* **2009**, *42*, 339-341.
3. Sheldrick, G. M. SHELXT – Integrated space-group and crystal structure determination. *Acta Cryst*. **2015**, *A71*, 3-8.
4. Sheldrick, G. M. Crystal structure refinement with SHELXL. *Acta Cryst.* **2015**, *C71*, 3-8.
